# A systematic review of the behaviour change techniques and digital features in technology-driven type 2 diabetes prevention interventions

**DOI:** 10.1177/2055207620914427

**Published:** 2020-03-24

**Authors:** Luke Van Rhoon, Molly Byrne, Eimear Morrissey, Jane Murphy, Jenny McSharry

**Affiliations:** 1 Health Behaviour Change Research Group, School of Psychology, National University of Ireland Galway, Ireland; 2 Medication Adherence Across the Lifespan Research Group, School of Psychology, National University of Ireland Galway, Ireland

**Keywords:** Systematic review, type 2 diabetes, diabetes prevention, diet, physical activity, digital health, health behaviour change, weight loss

## Abstract

**Objectives:**

Our aim was to conduct a systematic review to determine which technology-driven diabetes prevention interventions were effective in producing clinically significant weight loss, and to identify the behaviour change techniques and digital features frequently used in effective interventions.

**Methods:**

We searched five databases (CINAHL, EMBASE, MEDLINE, PsychINFO, and Pubmed) from inception to September 2018 and reviewed 19 experimental and non-experimental studies of 21 technology-driven diet plus physical activity interventions for adults (≥18 years) at risk of developing type 2 diabetes. Behaviour change techniques were coded using the BCT taxonomy v1, and digital features were identified via thematic analysis of intervention descriptions.

**Results:**

Sixty-three per cent of interventions were effective in the short term (achieving ≥3% weight loss at ≤6 months), using an average of 5.6 more behaviour change techniques than non-effective interventions, and 33% were effective in the long term (achieving ≥5% weight loss at ≥12 months), using 3.7 more behaviour change techniques than non-effective interventions. The techniques of social support (unspecified), goal setting (outcome/behaviour), feedback on behaviour, and self-monitoring of outcome(s) of behaviour were identified in over 90% of effective interventions. Interventions containing digital features that facilitated health and lifestyle education, behaviour/outcome tracking, and/or online health coaching were most effective.

**Conclusion:**

The integration of specific behaviour change techniques and digital features may optimise digital diabetes prevention interventions to achieve clinically significant weight loss. Additional research is needed to identify the mechanisms in which behaviour change techniques and digital features directly influence physical activity, dietary behaviours, and intervention engagement.

## Introduction

The global prevalence of diabetes represents a major public health concern. In 2015, the number of adults with diabetes was estimated at 415 million worldwide, with this figure projected to rise to 642 million by the year 2040.^1^ Type 2 diabetes (T2D) accounts for approximately 90% of all diabetes cases, and those with the condition face an additional two-to-fourfold risk of coronary heart disease.^2,3^ Being overweight and obese are the main drivers of T2D with 60% of diabetes cases directly attributed to weight gain.^4^ Based on international evidence from several landmark prevention studies,^5–7^ the International Diabetes Federation concluded that modifications to diet and physical activity are key to diabetes prevention.^3^ In the largest of these studies, the Diabetes Prevention Program included one-on-one health coaching and provided 16 30–60 minute educational sessions on diet, exercise, and behaviour modification. Participants lost an average of 5–7% of baseline body weight after 1 year, leading to a 58% study-wide reduction in T2D incidence over 3 years.^7^ Current diabetes prevention guidelines issued by the Centers for Disease Control and Prevention (CDC) in the USA, and the National Institute for Health and Care Excellence (NICE) in the UK, therefore recommend a weight loss target of at least 5%.^8,9^

Despite their effectiveness, the implementation of such large-scale, intensive programs may not be feasible in routine clinical practice where health care resources are limited.^10,11^ In view of this, smaller-scale diabetes prevention interventions (DPIs) have been adapted from the original Diabetes Prevention Program for implementation in ‘real world’ community settings.^12,13^ Systematic reviews of these community-based DPIs concluded that the interventions can promote clinically significant weight loss, as evidenced by an average 4–5% reduction in baseline body weight.^12,14^ However, despite offering greater accessibility and sustainability,^15^ community-based DPIs still require face-to-face delivery, which present participation barriers such as transportation, family/work commitments, and cost.^16,17^

Technology-driven DPIs have been developed to overcome the participation barriers of face-to-face DPIs by offering lifestyle education and support remotely or automatically via text messages, smartphone applications, or websites.^18^ Recent meta-analyses of DPIs delivered via digital technologies reported results comparable to the reviews of community-based DPIs. Bian et al.^19^ reported a mean 2-year weight loss of 4.81 kg across 15 studies, and Joiner, Nam, and Whittemore^20^ found an overall weight loss of 3.98% at 15 months across 22 studies. However, a number of the reviewed interventions were not necessarily technology-driven, with both meta-analyses including interventions that were delivered exclusively in real time by a human coach via phone or teleconference. Although these modes of delivery can be more accessible for participants, phone-based interventions require mutually convenient meeting times between participant and coach. Furthermore, these interventions may still incur substantial time and resource costs as health coaches must drive the intervention by frequently interacting with participants in real time. This may be particularly resource-intensive if sessions are delivered one-on-one. Importantly, both meta-analyses also reported significant inter-study heterogeneity in the modes of delivery, materials used, and the amount of weight lost, and the most effective behavioural and digital components or ‘active ingredients’ of the interventions remain unclear.

Behaviour change techniques (BCTs) are the observable, replicable, and irreducible intervention components, designed to modify the processes that regulate behaviour.^21^ A taxonomy of BCTs was developed to provide a standardised list of BCT labels and definitions, and evidence suggests that specific BCTs may be effective in improving dietary and physical activity behaviours.^21,22^ European diabetes prevention guidelines state that self-regulatory BCTs (e.g., goal setting, self-monitoring), action planning, problem solving, and social support should be present in all face-to-face DPIs.^10,23^ However, no review to date has assessed the use of BCTs in technology-driven DPIs.

Reviews of mobile health diabetes management studies have examined the links between technological features and intervention effectiveness. Donevant, Estrada, Culley, Habing, and Adams^24^ found that interventions with statistically significant outcomes used a combination of interactive features (where participants respond to or modify content in real time) and passive features (where a response is not required), while interventions without significant outcomes were more likely to have used passive features only. Holcombe^25^ found that interactive two-way text messages were more effective than passive one-way text messages at improving glycated haemoglobin (A1c) and medication adherence in adults with T2D. However, as the reviewed interventions focused on the management of T2D, it is not yet known which digital features are most effective in diabetes prevention. Furthermore, these reviews excluded interventions that were delivered using non-mobile digital platforms such as desktop computers or websites.

As DPIs that incorporate technology vary in content and outcomes, identifying the most effective behavioural and digital components in technology-driven DPIs is important to delineate potential causal pathways between components and outcomes and inform the cost and resource optimisation of future interventions. To achieve this, it must first be determined which technology-driven DPIs are effective in producing clinically significant weight loss and, following this, the most effective components can be identified. However, no review to date has either applied the BCT taxonomy to identify the techniques used in technology-driven DPIs, or performed a digital feature assessment. In light of this, the present review has two primary aims:

1. To determine which technology-driven DPIs were effective in producing clinically significant weight loss and improvements in additional outcomes linked to the onset of T2D.
2. To identify the BCTs and digital features most frequently used in effective interventions.

## Methods

This review followed the Preferred Reporting Items for Systematic Reviews and Meta-Analyses (PRISMA) guidelines Moher et al.^26^ (see Supplementary File 1). The protocol was registered with the International Prospective Register of Systematic Reviews (PROSPERO) [CRD42018097195].

### Study eligibility criteria

We included experimental and non-experimental studies, published in English, that assessed the effectiveness of technology-driven (e.g., automated phone calls or messages, smartphone application, text, email, instant message, video, website) diet and/or physical activity interventions for adults, age 18 and over, who are at risk of developing T2D (e.g., individuals with prediabetes, metabolic syndrome, overweight/obesity). This included observational studies, single-arm intervention studies, and randomised and non-randomised trials which assessed the intervention against a control group or alternative DPI. Studies must have had an explicit aim of preventing T2D or reducing the risk of developing T2D and reported at least one of the following outcomes: body weight, glycaemic status (either A1c or fasting glucose), or T2D incidence. Studies were excluded if: participants had previously received a diagnosis of type 1, type 2, or gestational diabetes; the interventions were delivered exclusively in real-time via a human coach (e.g., face-to-face, phone call, teleconferencing); or, if technology was only used to supplement an unmodified face-to-face intervention.

### Study search and selection

A systematic literature search of five databases (CINAHL, EMBASE, MEDLINE, PsycINFO, and PubMed) was conducted by the lead author (LV) to identify relevant studies published between database inception and 3 September 2018. Search terms (see Supplementary File 2) included key words, phrases, and Medical Subject Headings relevant to T2D risk, prevention interventions, diabetes-relevant outcomes, and digital modes of delivery.

All records retrieved from the database search were imported into EndNote X5^27^ and duplicates removed. All unique records were then imported into the Covidence software.^28^ Titles and abstracts were screened by one reviewer (LV) to determine potentially eligible full-text articles. The same reviewer screened all resulting full-text articles for inclusion. A second reviewer (JMu) independently screened a random 20% of the titles and abstracts, followed by a random 20% of the full-text articles. All initial disagreements were resolved via discussion between the two reviewers. Forward and backward reference searches of the included articles were then conducted by LV to identify additional articles.

### Outcomes and effectiveness assessment

The primary outcomes of interest were body weight, glycaemic status (A1c or fasting glucose), and T2D incidence. Body weight was chosen to inform this review’s primary definition of effectiveness as body weight has a strong association with T2D incidence, and is reported more often in DPI studies than the other primary outcomes.^4,11,19^ Intervention effectiveness was defined in relation to a mean weight loss of at least 5% of baseline body weight for two reasons. First, this figure is considered clinically significant^29^ and matches the US and UK weight loss benchmark for 12-month DPIs.^8,9^ Second, in the USA, for an organisation to receive accreditation as a certified Diabetes Prevention Program provider endorsed by the CDC, at least five participants must have completed the year-long programme, and the average weight loss after 12 months must have been at least 5%.^8^ Achieving this 5% has important implications as it can result in insurance coverage for participants and reimbursement for the organisations that deliver the programme.^30^

Interventions of ≤6 months were deemed effective if an average of ≥3% weight loss was achieved at ≤6 month follow-up, while interventions of ≥12 month duration were deemed effective if an average ≥5% weight loss was achieved at ≥12-month follow-up. Based on these criteria, interventions were labelled in four potential ways: (a) effective short term; (b) not effective short term; (c) effective long term; and, (d) not effective long term. Interventions of ≥12 month duration received two labels as they included short and long term follow-ups. Relationships were explored between the number and type of BCTs and digital features identified in effective versus non-effective interventions.

For the purpose of this review, BCTs and digital features were considered effective if they were identified in at least 75% of effective interventions, both short and long term. A BCT or digital feature was considered most effective at each respective time period (short or long term) if it was identified at considerably greater frequency in effective interventions compared to non-effective interventions.

### Data extraction

A data extraction tool was developed for this review and piloted on five randomly selected papers then refined and finalised. The extracted information included participant, study, and intervention characteristics, outcomes of absolute weight loss, percentage of baseline weight lost, A1c, fasting glucose, and T2D incidence – all of which were converted to standardised units where necessary. In cases where the average percentage of weight lost was not reported, this was hand calculated using the average baseline body weights and the average body weights at post intervention and subsequent follow-up(s). Data were extracted by one reviewer (LV), with a random 20% checked for accuracy by a second independent reviewer (EM). As the process of obtaining more detailed information from authors can take many months in which only a percentage of authors respond to such requests,^31^ only the publicly available materials (e.g., main study articles, follow-up study articles, intervention development articles, protocols, supplementary materials) pertaining to the included studies were used for data extraction, BCT coding, and digital feature identification.

#### Behaviour change technique coding

The BCT taxonomy v1^21^ was used by one reviewer (LV) to code BCTs from all intervention descriptions, and a second independent reviewer (EM) double coded a random 20% of all descriptions to check for reliability. All initial disagreements were resolved via discussion between the two reviewers. Based on previous reviews,^19,20^ it was anticipated that a number of different studies would describe the same standardised intervention such as those interventions based on the Diabetes Prevention Program. It was also anticipated that the interventions may be described differently in each study’s published literature where, for example, some BCTs clearly present in Study A’s intervention description(s) would be absent from Study B’s intervention description(s) and vice versa. To accommodate this, an imputation process was used to include the missing BCTs. First, intervention descriptions from each study were coded to identify the BCTs clearly present. Second, the BCTs coded as present in study A, but missing from study B, were also coded to Study B; the BCTs present in study B, but missing from study A, were coded to study A.

#### Digital feature identification

A modified three-phase thematic analysis^32^ was performed on all intervention descriptions to identify digital features. First, one reviewer (LV) analysed the descriptions, coding each digital component (e.g., nutrition video) and its mode of delivery (e.g., website), plus each non-digital component (e.g., food diary) and its format (e.g., hard copy). The aforementioned imputation process was also used to identify additional components in cases where multiple studies assessed the same standardised intervention. Second, digital components were categorised according to the level of interactivity between the participant and the digital tool and classified as either passive (one-way interaction) or interactive (two-way interaction). A second reviewer (EM) independently completed these first two phases on a random 20% of all intervention descriptions to check for reliability. Third, all passive and interactive digital components were pooled together in their respective groups and analysed by LV and EM via discussion. Through this discussion, common themes among the passive and interactive components were generated. These component clusters or themes were subsequently classified as either passive or interactive digital features and assigned labels that best represent each theme.

### Quality assessment

Study quality was assessed using the NICE quality appraisal checklist for quantitative intervention studies.^33^ This 27-item checklist enables appraisal of a study’s internal and external validity where each item is rated ++, +, or – based on the degree to which the criteria was satisfied, with ++ indicating highest quality or lowest risk of bias. One reviewer (LV) conducted the assessments and a random 20% were checked by a second reviewer (EM).

### Data synthesis

This review aimed to explore associations between two types of intervention components (BCTs and digital features) and the percentage of baseline weight lost and assess the effectiveness of interventions using international diabetes prevention benchmarks and certification requirements. Therefore, a narrative synthesis was chosen to organise and present the data within the text, with statistical data presented in the summary tables. As the majority of studies featured in the primary effectiveness analysis did not report the percentage of weight lost, sufficient data was not available for meta-analysis.

## Results

**Figure 1.**
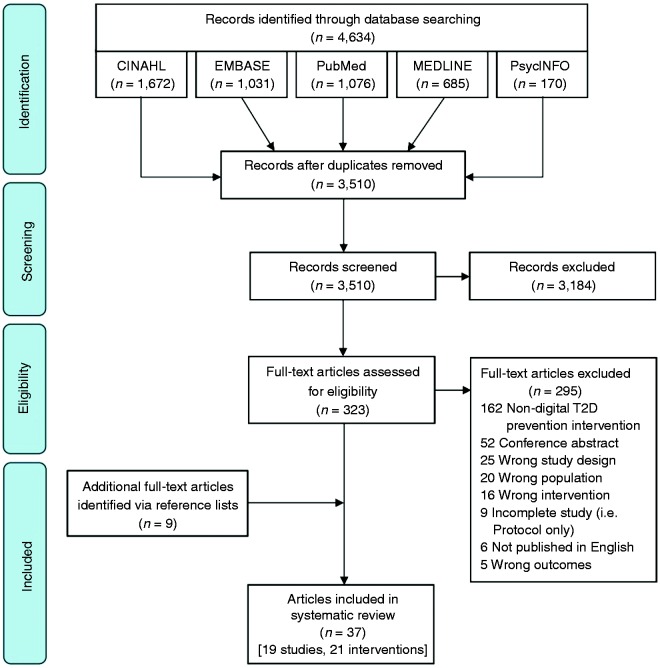
PRISMA flow diagram.

A total of 3510 unique articles were identified via electronic database searches (see Figure 1), with 323 remaining for full-text review. Following full-text review, 28 full-text articles were retained, and a forward and backward reference search identified nine additional articles. Thirty-seven articles (see Supplementary File 3) representing 19 studies of 21 interventions (two studies each assessed two unique technology-driven DPIs) were ultimately included. For studies reported in multiple articles, only the main article reporting the primary outcome measure(s) at first follow-up is referenced in the text and tables.

### Study characteristics

A summary of the characteristics of all 19 studies can be found in Table 1. Most studies (*n* = 14) were conducted in the USA,^34–47^ and the most common design (*n* = 10) was Randomised Controlled Trial.^34,37,39,40,42,46,48–51^ Study duration ranged between 3 months and 5 years, and enrolment was most often (*n* = 7) conducted in the primary care setting.^34,37,39–42,52^ The total number of intervention arm participants in the analyses was 2755 (65% female, age range 20–76 years). Two studies recruited males only,^49,51^ while the remainder recruited both males and females. Across the 10 studies which reported ethnicity in sufficient detail, 68% of participants were white. Across all intervention groups, short term attrition ranged between 9.4% and 43.4%, while the long term attrition range was 7.4% to 79.8%.

**Table 1.** Study characteristics.

Author(s)(year) Country*Intervention*	Study design	Comparison group(s)	Study duration	Enrolment setting	Definition of high risk of T2D	Sample (intervention group)	Attrition (intervention group)
Aguiar et al.(2016)Australia	Randomised Controlled Trial	Waitlist control	6 months	University	Australian Diabetes Risk Tool (AUSDRISK) score of ≥12.BMI: 25–40 kg/m^2^	*n* = 53Age range: 20–65 yearsMean age: 52.5 ± 9.5 yearsMale: 100%Ethnicity: not reportedMean BMI: 32.2 ± 3.5 kg/m^2^	9.4% at 3 months24.5% at 6 months
Arens et al. (2018)Germany	Prospective observational study	Usual care	12 months	Primary care	Presence of metabolic syndrome.	*n* = 109Age range: 35–60 yearsMean age: 49.6 ± 9.3 yearsFemale: 60.6%Ethnicity: not reportedMean BMI: 32.2 ± 5.5 kg/m^2^	19.3% at 3 months32.1% at 6 months49.5% at 9 months79.8% at 12 months
Block et al.(2015)USA	Randomised Controlled Trial	Waitlist control	6 months	Primary care	Presence of prediabetesBMI: ≥27 kg/m^2^ (≥25 kg/m^2^ for Asian subgroups).Fasting glucose: 100–125 mg/dLA1c: 5.7–6.4%.	*n* = 163Age range: 31–70 yearsMean age: 55 ± 8.8 yearsMale: 68.1%White: 66.9%Mean BMI: 31.1 ± 4.5 kg/m^2^	16.6% at 6 months
Castro Sweet et al. (2018)USA	Single-arm prospective study	NA	12 months	Online	Presence of prediabetes (A1c: 5.7–6.4%). Metabolic syndrome (Prediabetes, hypertension, dyslipidaemia and obesity).	*n* = 501Age range: not reportedMean age: 68.8 ± 2.6 yearsFemale: 64%White: 60.3%Mean BMI: 33.6 ± 5.7 kg/m^2^	4% of participants did not meet CDC DPRP criteria (as they completed ≤3 intensive phase lessons).
Cha et al.(2014)USA	Single-arm prospective pilot study	NA	12 weeks	University	Presence of prediabetes (impaired fasting glucose: 100–125 mg/dL; or, A1c: 5.7–6.4%).	Intervention completers:*n* = 13Age range: 21–28 yearsMean age: 24.4 ± 2.2 yearsFemale: 76.9%African American: 53.8%Mean BMI: not reported	13.3% at 12 weeks
Estabrooks and Smith-Ray (2008)USA	Randomised Controlled Trial	Usual care	3 months	Primary care	Elevated blood glucose and/or clinical diagnosis of prediabetes.	*n* = 39Age range: not reportedMean age: 57.8 ± 17 yearsFemale: 71.8%White: 69%Mean BMI: not reported	28.2% at 3 months
Everett et al. (2018)USA	Single-arm prospective observational study	Calibration cohort	3 months	University hospital	Diagnosis of prediabetes (fasting glucose: 100–125 mg/dL; impaired glucose tolerance: 2-hour glucose of 140–199 mg/dL after 75g oral glucose tolerance test; or, A1c: 5.7–6.4%).BMI: 24–40 kg/m2 (22–40 kg/m2 for Asian individuals).	Intervention completers only:*n* = 38Age range: not reportedMean age: 57.2 ± 9.1 yearsFemale: 63%White: 82%Mean BMI: not reported	11.6% at 3 months
Fischer et al. (2016)USA	Randomised Controlled Trial	Usual care	12 months	Primary care	A1c between 5.7% and 6.4%.	*n* = 78Age range: not reportedMean age: 47.7 ± 12.4Female: 70.5%Native Spanish speakers: 65%Mean BMI: not reported	7.7% at 12 months
Fukuoka et al.(2015)USA	Feasibility Randomised Controlled Trial	Pedometer only control	5 months	Primary care	BMI: ≥25 kg/m^2^ (22 kg/m^2^ if Asian-Pacific Islander).American Diabetes Association Diabetes Risk Test score of ≥5. Fasting plasma glucose: 100–125 mg/dL; A1c: 5.7–6.4%; Oral glucose tolerance test: 140–200 mg/dL.	*n* = 30Age range: 36–76 yearsMean age: 57.1 ± 9.1 yearsFemale: 76.7%White: 43.3%Mean BMI: 32.2 ± 5.6 kg/m^2^	10% of participants did not complete 3-month follow-up assessment. 6.6% of participants did not complete 5-month follow-up assessment.
Kramer et al. (2010)USA	Non-randomised Controlled Trial	Face-to-face intervention	3 months	Primary care	BMI ≥25 kg/m^2^Prediabetes (Fasting glucose: 100–125 mg/dL).Presence of metabolic syndrome.	*n* = 22Age range: not reportedMean age: 57.3 yearsSex/gender: not reportedEthnicity: not reportedMean BMI: 32.9 ± 6.1 kg/m^2^	36.4% at 3 months
Limaye et al. (2017)India	Randomised Controlled Trial	Standard care	12 months	Worksite	Presence of ≥3 risk factors (family history of cardio-metabolic disease, overweight/obesity, high blood pressure, impaired fasting glucose, Hypertriglyceridaemia, high LDL and low HDL cholesterol).	*n* = 133Age range: not reportedMean age: 36.8 ± 7.2 yearsMale: 74.4%Ethnicity: not reportedMean BMI: 27 ± 3.2 kg/m^2^	21.1% at 12 months
Ma et al. (2013)USA	Randomised Controlled Trial	Coach-led intervention; usual care control	15 months	Primary care	BMI: ≥25 kg/m^2^Prediabetes (fasting plasma glucose: 100–125 mg/dL) Metabolic syndrome (central obesity, dyslipidaemia, hypertension, prediabetes).	*n* = 81Age range: not reportedMean age: 51.8 ± 9.9 yearsMale: 54.3%White: 79%Mean BMI: 31.7 ± 4.7 kg/m^2^	7.4% at 15 months
Michaelides et al. (2016)USA	Single-arm prospective study	NA	24 weeks(plus 65 week follow-up)	Worksite	Hyperglycaemia (A1c: 5.7–6.4%).	Program starters: *n* = 43Age range: not reportedMean Age: 51.5 ± 8.3 yearsFemale: 86%Ethnicity: not reportedMean BMI: 35.5 ± 7.4 kg/m^2^	16.3% of program starters (read >1 article per week for ≥4 weeks) did not complete the core program.
Piatt et al.(2013)USA*GLB-DVD*	Prospective quasi-experimental study	Face-to-face; internet; self-selection interventions	6 months(plus 18 month follow-up)	University	BMI: ≥25 kg/m^2^Abdominally obese (waist circumference: >40 inches in males and >25 inches in females).	*n* = 113Age range: not reportedMean age: 52.4 ± 10.9 yearsFemale: 85%White: 93.8%Mean BMI: 36.2 ± 7.2 kg/m^2^	43.4% at 6 months
Piatt et al.(2013)USA*GLB-Internet*	Prospective quasi-experimental study	Face-to-face; DVD; self-selection interventions	6 months(plus 18 month follow-up)	University	BMI: ≥25 kg/m^2^Abdominally obese (waist circumference: >40 inches in males and >25 inches in females).	*n* = 101Age range: not reportedMean age: 48.7 ± 9.7 yearsFemale: 88.1%White: 99.1%Mean BMI: 36.1 ± 6.4 kg/m^2^	56.4% at 6 months
Ramachandran et al. (2013)India	Randomised Controlled Trial	Usual care	24 months(plus five year follow-up)	Worksite	Positive family history of T2D.BMI: ≥23 kg/m^2^	*n* = 271Age range: not reportedMean age: 54.1 ± 6.1 yearsMale: 100%Ethnicity: not reportedMean BMI: 25.8 ± 3.3 kg/m^2^	3.7% at 24 months
Sepah et al. (2014)USA	Quasi-experimental Single-arm prospective study	NA	12 months (plus 24 and 36 month follow-ups)	Online	BMI: ≥25 kg/m^2^ (22 kg/m^2^ if Asian).	Core group:*n* = 187Age range: not reportedMean age: 43.9 ± 12.4 yearsFemale: 85%White: 51%Mean BMI: 36.7 ± 7.6 kg/m^2^	15% of participants did not meet CDC DPRP ‘core phase’ criteria (as they only completed ≤3 core lessons). 34.5% did not meet ‘post-core phase’ criteria (completed ≤3 core lessons and 0 post-core lessons)
Tate et al.(2003)USA*Basic Internet*	Randomised Controlled Trial	Internet and Behavioural e-Counselling Intervention	12 months	University hospital	BMI between 27–40 kg/m^2^≥1 risk factors for T2D (e.g., family history of T2D, impaired glucose tolerance).	*n* = 46Age range: not reportedMean age: 47.3 ± 9.5 yearsFemale: 89%White: 89%Mean BMI: 33.7 ± 3.7 kg/m^2^	15.2% at 12 months
Tate et al.(2003)USA*Internet and Behavioral e-Counseling*	Randomised Controlled Trial	Basic Internet Intervention	12 months	University hospital	BMI between 27–40 kg/m^2^≥1 risk factors for T2D (e.g., family history of T2D, impaired glucose tolerance).	*n* = 46Age range: not reportedMean age: 49.8 ± 9.3 yearsFemale: 91%White: 89%Mean BMI: 32.5 ± 3.8 kg/m^2^	17.4% at 12 months
Wilson et al.(2017)USA	Non-randomised controlled observational study	Matched control	2 years	Worksite	BMI: ≥24 kg/m^2^ (22 kg/m^2^ if Asian); Prediabetes (fasting blood glucose: 100–125 mg/dL, A1c: 5.7–6.4%, oral glucose tolerance test: 140–199 mg/dL).	*n* = 634Age range: 23–68 yearsMedian age: 46 yearsFemale: 58.4%White: 68%Mean BMI: 34.5 kg/m^2^	5.8% of participants did not meet CDC DPRP criteria (completed ≤3 intensive phase lessons). 76% of participants had sufficient data for analysis.
Wong et al. (2013)Hong Kong	Randomised Controlled Trial	Usual care	24 months	University hospital	Diagnosis of prediabetes (fasting plasma glucose: 5.6–6.9 mmol/L; or, 2-hour postprandial glucose: 7.8–11.0 mmol/L after 75g glucose load).	*n* = 54Age range: not reportedMean age: 54.1 ± 6.1 yearsMale: 90.7%Ethnicity: not reportedMean BMI: 25.6 ± 2.9 kg/m^2^	16.7% at 12 months24.1% at 24 months

NA: Not Applicable.

### Intervention characteristics

A summary of the characteristics from all 21 technology-driven DPIs can be found in Table 2. The intervention delivery period ranged between 3 and 24 months in duration, and all interventions targeted both diet and physical activity behaviours. Eleven interventions were independent (newly developed),^34,36–38,46,48–52^ and 10 were largely adapted from a previous face-to-face program. Of these 10, six^35,39,40,43,45,47^ were adapted from the Diabetes Prevention Program,^53^ and four^41,42,44^ were adapted from the Group Lifestyle Balance Program.^54^ Sixteen interventions were informed by at least one theory or framework, with Social Cognitive Theory (*n* = 14) the most common. Digital modes of delivery included: website;^34–36,44–47,51^ smartphone app;^35,36,38,40,43,45,47,52^ DVD;^41,42,44,51^ SMS;^39,48–50^ email;^34,46,48^ and, Interactive Voice Response.^34,37^ Eight interventions used multiple digital modes of delivery.^34–36,45–48,51^ Nine interventions were ‘stand-alone’ as they did not include human health coach support.^34,37,38,42,46,48–51^ Of the 12 interventions with health coach support, nine incorporated remote online or phone support,^35,36,41,43–47^ one incorporated face-to-face support,^40^ and two included both remote and face-to-face support.^39,52^

**Table 2.** Intervention characteristics.

Author(s)(year)	Intervention name	Intervention duration	Intervention type	Primary mode(s) of delivery	Level of support	Theoretical basis	Message content and frequency
Aguiar et al.(2016)	PULSE	6 months	Independent	Website and DVD	Stand alone	Social Cognitive Theory	The PULSE Program was entirely self-paced and included the (also self-paced) Self-Help, Exercise and Diet Using Internet Technology (SHED-IT) weight loss program for men.
Arens et al. (2018)	NA	12 months	Independent	Smartphone application	Remote and face-to-face support via physician	NR	Participants were to regularly enter weight, abdominal girth, blood pressure, and blood glucose into the app. Participants were invited to attend up to nine classes on nutrition and physical activity. Via a web-portal, physicians provided participants with regular feedback, messages, and goal modification.
Block et al.(2015)	Alive-PD	6 months	Independent	Website, Interactive Voice Response, and Email	Stand alone	Learning Theory; Social Cognitive Theory; Theory of Planned Behaviour	The Alive-PD was self-administered. Two weekly health notes provided health information. Participants engaged in weekly tailored goal setting and tracking. Individually tailored print materials were sent monthly. Automated individually tailored phone coaching was delivered every two weeks via Interactive Voice Response.
Castro Sweet et al. (2018)	Omada Health Program	12 months (16 week intensive + 36 week maintenance)	Diabetes Prevention Program	Website and smartphone application	Online support via health coach	Social Cognitive Theory; Transtheoretical model	For the initial 16-week intensive weight loss phase, participants completed one 1-hour online lesson each week. Less frequent lessons were completed in the subsequent 36-week weight maintenance phase. Participants engaged with their health coach and other participants online throughout the 12-month program.
Cha et al.(2014)	NA	12 weeks	Independent	Website and smartphone application	Remote phone support via undergraduate student	Social Cognitive Theory; AADE7 Self-Care Behaviors Framework	Participants submitted weekly dietary and exercise habits and biweekly assignments. An undergraduate student on the research team provided weekly script-based phone counselling sessions.
Estabrooks and Smith-Ray(2008)	NA	3 months	Independent	Interactive Voice Response	Stand alone	NR	Automated calls delivered once per week for 12 weeks. Seven calls provided 5–10 minutes of counselling and the remaining five calls provided a tip of the week.
Everett et al. (2018)	Sweetch Mobile Intervention	3 months	Independent	Smartphone application	Stand alone	Just-in-time adaptive intervention design	The Sweetch app used machine learning to present users with content based on their own real-world life habits. Message content and frequency varied between users.
Fischer et al. (2016)	NA	12 months	Diabetes Prevention Program	Short Message Service (SMS)	Face-to-face and phone support via health coach, and nutritionist or nurse.	Social Cognitive Theory; Transtheoretical model	Participants received six text messages per week and were prompted to report their weight once per week. Participants were eligible for motivational interviewing phone appointments with a health coach, in addition to weight loss resources such as access to DPP classes and appointments with a nutritionist or nurse for diet support.
Fukuoka et al.(2015)	mDPP	5 months	Diabetes Prevention Program	Smartphone application	Face-to-face support via non-medical research staff	Social Cognitive Theory; Transtheoretical model	The mobile app delivered daily messages, video clips, and quizzes. Participants attended six in-person sessions within a 4-month period.
Kramer et al. (2010)	GLB-DVD	3 months	Group Lifestyle Balance	DVD	Remote phone support via health care professional	Social Cognitive Theory; Transtheoretical model	Participants viewed one DVD per week. Participants contacted by health care professional once per week to review performance and voice questions/concerns.
Limaye et al. (2017)	LIMIT (Lifestyle modification in IT)	12 months	Independent	Short Message Service (SMS) and Email	Stand alone	NR	Participants received lifestyle modification information via mobile phone and e-mail for one year. Three mobile phone messages and two e-mails were sent per week. A total of 150 phone messages and 100 emails were sent to each participant during the intervention period.
Ma et al. (2013)	E-LITE	15 months (3 month intensive + 12 month maintenance)	Group Lifestyle Balance	DVD	Stand Alone	Social Cognitive Theory; Transtheoretical model	In the intensive treatment phase, participants were instructed to watch one DVD session per week for 12 weeks. In the maintenance phase, participants received an email reminder every two weeks to continue self-monitoring.
Michaelides et al. (2016)	Noom Coach	24 weeks (16 week core + 8 week post-core)	Diabetes Prevention Program	Smartphone application	Remote app-based support via health coach	Social Cognitive Theory; Transtheoretical model	Participants received daily articles and interactive challenges and log their weight, meals, and physical activity each week into the app. The health coach communicated with participants twice per month.
Piatt et al.(2013)	GLB-DVD	12–14 weeks	Group Lifestyle Balance	DVD	Phone support via registered nurse or dietician	Social Cognitive Theory; Transtheoretical model	Participants instructed to watch one DVD session per week for 12 weeks. Participants also met as a group at four time points within the 12-week period. Preventionists and lay health coaches called participants weekly to offer information and support.
Piatt et al.(2013)	GLB-Internet	12–14 weeks	Group Lifestyle Balance	Website	Online counselling via registered nurse or dietician	Social Cognitive Theory; Transtheoretical model	Participants were instructed to watch one video per week for 12 weeks. Participants also met as a group at baseline and again after completing the intervention. Preventionists and lay health coaches supported participants via online counselling.
Ramachandran et al. (2013)	NA	24 months	Independent	Short Message Service (SMS)	Stand alone	Transtheoretical Model	Participants received 2–4 text messages per week for 24 months. Messages contained <160 characters.
Sepah et al. (2014)	Prevent (Omada Health Program)	12 months (16 week core + 36 week post-core)	Diabetes Prevention Program	Website and smartphone application	Online support via health coach	Social Cognitive Theory; Transtheoretical model	Participants were matched into online groups of 10 to 15 people and communicated via online social network. In the 16-week core phase, participants completed 16 weekly online lessons. In the 12-month post-core phase, participants completed 9 monthly lessons.
Tate et al.(2003)	Basic Internet	12 months	Independent	Website	Stand alone	NR	Weekly weight loss tutorials and tips were delivered via website. Participants were sent weekly email reminders to submit weight.
Tate et al.(2003)	Internet and Behavioral e-Counseling	12 months	Independent	Website and Email	Remote e-mail support via counsellor	NR	Weekly weight-loss tutorials and tips were delivered via website. Participants were sent weekly email reminders to submit weight. The counsellor emailed participants five times during the first month and weekly for the remaining 11 months.
Wilson et al.(2017)	Omada Health Program	12 months (16 week core + 36 week post-core)	Diabetes Prevention Program	Website and smartphone application	Online support via health coach	Social Cognitive Theory; Transtheoretical model	For the initial 16-week intensive weight loss phase, participants completed one lesson each week. Participants completed additional weekly lessons during the subsequent 36-week weight maintenance phase. Participants engaged with their health coach and other participants online throughout the 12-month program.
Wong et al. (2013)	NA	24 months	Independent	Short Message Service (SMS)	Stand alone	Social Cognitive Theory; Theory of Planned Behaviour	Phase 1: three text messages per week (36 total)Phase 2: one text per week (12 total)Phase 3: one text per month (6 total)Phase 4: one text per month (12 total)

*Note:* NA: not applicable; NR: not reported.

### Quality assessment

A summary of the quality assessments for all 19 studies can be found in Supplementary Table 1. Fifteen studies (all 10 RCTs and five of the nine non-RCTs) achieved a high quality rating for internal validity through minimisation of bias across multiple criteria. Ten studies (seven of the 10 RCTs and three of the nine non-RCTs) achieved a high quality rating for external validity, with findings generalisable to the source population.

### Intervention effectiveness

Two studies were excluded from the primary effectiveness assessment. The study by Arens, Hauth, and Weissmann^52^ was excluded as they implemented rolling follow-ups where a common intervention end point could not be determined. However, on average, participants remained in the intervention for 8.3 months, losing 2.4 kg (*SD* = 6.3, *p* < .0001). The study by Ramachandran et al.^49^ was excluded as body weight was not a key outcome and therefore not reported. The range of weight lost across the remaining 19 interventions was 0.69% to 8% in the short term and 0.93% to 7.5% in the long term (see Supplementary Table 2).

Based on this review’s primary effectiveness criteria, 12 interventions were effective short term,^34,35,40–47,51^ these included both the GLB-DVD and GLB-Internet interventions by Piatt et al.^44^ and the Behavioural e-Counseling intervention by Tate et al.^46^ Seven interventions were not effective short term,^36–39,46,48,50^ including the Basic Internet intervention by Tate et al.^46^ Four interventions were effective long term,^35,42–44^ including the GLB-Internet intervention by Piatt et al.^44^ Eight interventions were not effective long term;^39,44–48,50^ including the GLB-DVD intervention by Piatt et al.^44^ and both the Behavioural e-Counseling and Basic Internet interventions by Tate et al.^46^

Of the four interventions that included an active weight maintenance phase (8–12 months in duration) with sufficient outcome data, one achieved further weight loss^43^ and three achieved weight maintenance (as indicated by <0.5% change in body weight) during this period.^35,42,45^ Four interventions included follow-ups that were conducted 12 or more months after the intervention was complete. Of these, both the GLB-DVD and GLB-Internet interventions by Piatt et al.^44^ achieved further weight loss at 12 months post-intervention, one achieved weight maintenance at 12 months,^43^ and one achieved weight maintenance at 12 months but reported a 39% regain of lost weight at 24 months.^45^

### Secondary measures

#### Change in glycaemia

Complete results for changes in A1c and fasting glucose were reported for 9 and 13 interventions respectively (see Supplementary Table 3). Seven interventions achieved significant improvement in A1c^34–36,38,41,45,51^ and five interventions achieved significant improvement in fasting glucose.^34,41,42,47,48^

#### Incidence of T2D

Incidence rates for T2D were reported for two interventions. Wong et al.^50^ found a 24-month T2D incidence rate of 11.11% and 18% in the intervention and usual care groups respectively. However, this difference was not significant. Ramachandran et al.^49^ reported significantly lower T2D incidence, HR = 0.700, *p* = .009, 95% CI = (0.53, 0.93), among the intervention group (18%, and 33.9% at 24 and 60 months respectively) compared to the usual care group (27%, and 44.9% at 24 and 60 months respectively).

### Behaviour change techniques

Thirty unique BCTs were coded from all 21 interventions (see Supplementary Table 4), with an average of nine BCTs per intervention (range: 1–14). A summary of the BCTs identified in effective and non-effective interventions can be found in Table 3. Seven BCTs were identified in at least 75% of effective interventions, both short and long term. These were: goal setting (behaviour) (identified in 92% and 100% of effective interventions in the short and long term respectively), problem solving (75% and 100%), goal setting (outcome) (92% and 100%), feedback on behaviour (92% and 100%), self-monitoring of behaviour (92% and 75%), self-monitoring of outcome(s) of behaviour (92% and 100%), and social support (unspecified) (100% and 100%).

**Table 3.** Summary of behaviour change technique use in effective and non-effective interventions.

		All interventions(*N* = 21)		Effective ST(*N* = 12)		Not effective ST(*N* = 7)		Effective LT(*N* = 4)		Not-effective LT(*N* = 8)	
No.	Behaviour change technique	*n*	%	*n*	%	*n*	%	*n*	%	*n*	%
Cluster One: Goals and planning											
1.1	Goal setting (behaviour)	16	76.2	11	91.7	4	57.1	4	100	5	62.5
1.2	Problem solving	14	66.7	9	75	3	42.9	4	100	4	50
1.3	Goal setting (outcome)	15	71.4	11	91.7	3	42.9	4	100	5	62.5
1.4	Action planning	7	33.3	6	50	1	14.3	2	50	1	12.5
1.5	Review behaviour goals	5	23.8	4	33.3	1	14.3	2	50	1	12.5
1.7	Review outcome goals	4	19	4	33.3	0	0	2	50	1	12.5
Cluster Two: Feedback and monitoring											
2.2	Feedback on behaviour	15	71.4	11	91.7	3	42.9	4	100	5	62.5
2.3	Self-monitoring of behaviour	16	76.2	11	91.7	4	57.1	3	75	6	75
2.4	Self-monitoring of outcome(s) of behaviour	15	71.4	11	91.7	3	42.9	4	100	6	75
2.7	Feedback on outcome(s) of behaviour	1	4.8	0	0	0	0	0	0	0	0
Cluster Three: Social support											
3.1	Social support (unspecified)	14	66.7	12	100	2	28.6	4	100	6	75
3.2	Social support (practical)	1	4.8	0	0	1	14.3	0	0	1	12.5
3.3	Social support (emotional)	6	28.6	5	41.7	1	14.3	2	50	3	37.5
Cluster Four: Shaping knowledge											
4.1	Instruction on how to perform the behaviour	4	19	1	8.3	1	14.3	0	0	1	12.5
4.2	Information about antecedents	6	28.6	5	41.7	1	14.3	2	50	3	37.5
Cluster Five: Natural consequences											
5.1	Information about health consequences	5	23.8	2	16.7	2	28.6	0	0	2	25
Cluster Six: Comparison of behaviour											
6.1	Demonstration of the behaviour	2	9.5	1	8.3	1	14.3	0	0	0	0
6.2	Social comparison	7	33.3	6	50	1	14.3	2	50	3	37.5
Cluster Seven: Associations											
7.1	Prompts/cues	5	23.8	4	33.3	1	14.3	2	50	1	12.5
Cluster Eight: Repetition and substitution											
8.2	Behaviour substitution	3	14.3	1	8.3	1	14.3	0	0	1	12.5
8.3	Habit formation	2	9.5	2	16.7	0	0	0	0	0	0
8.4	Habit reversal	1	4.8	1	8.3	0	0	0	0	0	0
8.7	Graded tasks	1	4.8	1	8.3	0	0	0	0	0	0
Cluster Nine: Comparison of outcomes											
9.1	Credible source	7	33.3	5	41.7	1	14.3	2	50	2	25
Cluster Ten: Reward and threat											
10.1	Material incentive (behaviour)	1	4.8	1	8.3	0	0	0	0	0	0
10.2	Material reward (behaviour)	1	4.8	1	8.3	0	0	0	0	0	0
Cluster Eleven: Regulation											
11.2	Reduce negative emotions	3	14.3	1	8.3	1	14.3	0	0	1	12.5
Cluster Twelve: Antecedents											
12.3	Avoidance/reducing exposure to cues for the behaviour	1	4.8	0	0	1	14.3	0	0	1	12.5
12.5	Adding objects to the environment	9	42.9	8	66.7	0	0	3	75	3	37.5
Cluster Fourteen: Scheduled consequences											
14.4	Reward approximation	1	4.8	0	0	1	14.3	0	0	0	0
Average number of BCTs per intervention		9		11.3		5.4		11.5		7.8	

*Note:* ST: short term (≤6 month) follow-up; LT: long term (≥12 month) follow-up; *N*: number of interventions; *n*: number of interventions in which the BCT was identified; %: proportion of interventions that used the BCT.

#### Short term effectiveness

Interventions that achieved short term effectiveness used an average of 11.3 BCTs (range: 4–14), compared to 5.4 (range: 1–10) among non-effective interventions. Two BCTs were identified at a considerably greater frequency in effective interventions versus non-effective interventions. These were social support (unspecified) (identified in 100% of effective interventions versus 29% of non-effective interventions) and adding objects to the environment – coded when participants were issued pedometers to count their steps (67% versus 0%).

#### Long term effectiveness

Interventions that achieved long term effectiveness used an average of 11.5 BCTs (range: 10–13), compared to 7.8 (range: 1–13) among non-effective interventions. One BCT, problem solving, was identified at a considerably greater frequency in effective interventions versus non-effective interventions (100% versus 50%).

### Digital features

The digital and non-digital components coded from all 21 interventions can be found in Supplementary File 5. Ten digital features – five passive and five interactive (see Supplementary Table 5) – were identified via thematic analysis of intervention descriptions. Detailed descriptions of all ten digital features can be found in Supplementary File 6. The five passive features were: health and lifestyle information and advice; activity tracking; reminders and prompts; diet tracking; and, weight and biomeasure tracking. The five interactive features were: interactive health and lifestyle lessons; social media and support; online health coaching; automated feedback; and gamification. Interventions used an average of 4.3 digital features (range: 1–9), including 2.9 passive features (range: 1–5) and 1.4 interactive features (range: 0–4).

A summary of the digital features identified in effective and non-effective interventions can be found in Table 4. Three digital features (all passive) were identified in at least 75% of effective interventions, both short and long term. These were: activity tracking (identified in 100% and 100% of effective interventions in the short and long term respectively), health and lifestyle information and advice (75% and 75%); and diet tracking (75% and 75%). It is noteworthy that the interactive social media and support feature was identified in only 50% of the effective interventions, yet the social support (unspecified) BCT was identified in 100% of effective interventions. Additionally, of the three interventions that only used paper based rather than digital tools to track diet and physical activity, two were not effective in the short term, and all three were not effective in the long term (data not shown).

**Table 4.** Summary of digital feature use in effective and non-effective interventions.

Digital features	All interventions(*N* = 21)		Effective ST(*N* = 12)		Not effective ST(*N* = 7)		Effective LT(*N* = 4)		Not effective LT(*N* = 8)	
	*n*	%	*n*	%	*n*	%	*n*	%	*n*	%
Passive features										
Health and lifestyle information and advice	16	76.2	9	75	6	85.7	3	75	6	75
Activity tracking	15	71.4	12	100	2	28.6	4	100	4	50
Reminders and prompts	11	52.4	8	66.7	3	42.9	4	100	4	50
Diet tracking	10	47.6	9	75	1	14.3	3	75	3	37.5
Weight and biomeasure tracking	9	42.9	7	58.3	1	14.3	3	75	2	25
Average passive features per intervention	2.9 features		3.75 features		1.86 features		4.25 features		2.38 features	
Interactive features										
Interactive health and lifestyle lessons	9	42.9	6	50	2	28.6	1	25	4	50
Social media and support	8	38.1	6	50	2	28.6	2	50	5	62.5
Online health coaching	8	38.1	7	58.3	0	0	4	100	3	37.5
Automated feedback	4	19	2	16.7	2	28.6	0	0	0	0
Gamification	1	4.8	1	8.3	0	0	0	0	0	0
Average interactive features per intervention	1.43 features		1.83 features		0.86 features		1.75 features		1.5 features	
Average total features per intervention	4.3		5.58		2.71		6		3.88	

*Note:* ST: short term (≤6 month) follow-up; LT: long term (≥12 month) follow-up. *N*: number of interventions; *n*: number of interventions in which the feature was identified; %: proportion of interventions that used the digital feature.

#### Short term effectiveness

Interventions that achieved short term effectiveness used an average of 5.6 total features (range: 3–9), including 3.8 passive features (range: 2–5) and 1.8 interactive features (range: 0–4). Non-effective interventions used an average of 2.7 total features (range: 1–5), including 1.9 passive features (range 1–4) and 0.9 interactive features (range: 0–2). Three digital features were identified at a considerably greater frequency in effective interventions versus non-effective interventions. These were the passive features of activity tracking (identified in 100% of effective interventions versus 29% of non-effective interventions) and diet tracking (75% versus 14%), and the interactive feature of online health coaching (58% versus 0%).

#### Long term effectiveness

Interventions that achieved long term effectiveness used an average of 6 total features (range: 4–7), including 4.3 passive features (range: 3–5) and, 1.8 interactive features (range: 1–3). Non-effective interventions used an average of 3.9 total features (range: 1–7), including 2.4 passive features (range: 1–4) and 1.5 interactive features (range: 1–4). Four digital features were identified at a considerably greater frequency in effective interventions versus non-effective interventions. These were the passive features of activity tracking (100% versus 50%), reminders and prompts (100% versus 50%), weight and biomeasure tracking (75% versus 25%), and the interactive feature of online health coaching (100% versus 38%).

### Additional analyses

As the imputation process used in this review is a novel means of coding BCTs and digital components, additional analyses were conducted using only those BCTs and digital components clearly present in each study’s intervention description(s). Results of these analyses, which exclude any BCT or digital feature coded via imputation, can be found in Supplementary Tables 8–10.

## Discussion

This systematic review assessed 19 studies of 21 technology-driven DPIs, with the aims of determining which interventions were effective in producing clinically significant weight loss and improvements in additional outcomes linked to the onset of T2D and identifying the most effective BCTs and digital features. This review found that, in the short term (≤6 months), most technology-driven DPIs successfully achieved clinically significant weight loss in adults at risk of developing T2D, as determined by an average weight loss of at least 3% of baseline body weight. However, most interventions fell short of achieving the 5% weight loss benchmark for clinical significance at ≥12 months. Follow-up data indicated that weight loss was maintained for at least one year post-intervention. Additionally, seven and five interventions achieved significant improvements in A1c and fasting glucose respectively, and one study found a significantly lower 5-year incidence of T2D among participants who completed the intervention compared to those who received usual care – evidence to support the effectiveness of technology-driven DPIs in diabetes prevention. Across all the reviewed studies, there was wide heterogeneity in study populations, attrition rates, intervention duration, and mode of delivery. Comparable findings on the effectiveness of technology-driven DPIs and inter-study heterogeneity were reported in previous meta-analyses.^19,20^

### Behaviour change techniques

Interventions which used a larger number of BCTs were more effective. This is consistent with reviews of face-to-face interventions for individuals with T2D^55–57^ or those at risk of developing T2D.^58^ Seven unique BCTs were frequently identified in effective interventions. These were: social support (unspecified), goal setting (behaviour), goal setting (outcome), feedback on behaviour, self-monitoring of outcome(s) of behaviour, self-monitoring of behaviour, and problem solving. All of these BCTs correspond to the recommended behaviour change components for face-to-face DPIs as outlined in the IMAGE toolkit for the prevention of T2D in Europe.^23^ Therefore, the present findings suggest these recommendations should extend to technology-driven DPIs. Of the recommended behaviour change components described in the toolkit, action planning was the only corresponding BCT that was not identified in at least 75% of effective interventions. Nevertheless, as action planning was identified more frequently in effective than non-effective interventions in both the short and long term, this technique may still be a valuable inclusion in technology-driven DPIs.

In the short term, effective interventions used, on average, 5.6 more BCTs than non-effective interventions, with social support (unspecified) and adding objects to the environment the most effective BCTs. A number of digital social support-based weight loss interventions have reported significant weight loss.^59–61^ However, the broad nature of the social support (unspecified) BCT may have increased the frequency in which it was coded in the present review relative to other BCTs. As this BCT accommodates a wide range of social support strategies, a rationale for the effectiveness of social support in the present review is difficult to discern, and weight loss may have occurred via interactions between social support and other intervention components. Furthermore, studies of online weight loss communities found weight loss or weight gain to depend on: the type(s) of social support available; how participants provided and received support; and the level in which participants engaged with the support opportunities.^62–65^ Therefore, for a nuanced understanding of the relationship between social support and weight loss in technology-driven DPIs, further assessment is needed to identify the perceptions and experiences of participants who engaged or disengaged with the social support tools and opportunities. Adding to the success of social support, all eight interventions that issued pedometers were effective – perhaps unsurprising given that pedometer-based walking interventions, even those lacking dietary intervention, have achieved modest weight loss.^66^ However, weight loss may not be the product of pedometer use per se, as goal setting (e.g., daily step targets) could have motivated participants to increase their physical activity to the level required for weight reduction. Supporting this, a review of pedometer use among adult outpatients reported a 27% increase in physical activity and significant decrease in Body Mass Index (BMI), with goal setting the key outcome predictor.^67^ It is also possible that participants perceived the self-contained pedometer to be a practical gift of value, providing an incentive to engage with the intervention in its early stages.

In the long term, effective interventions used, on average, 3.7 more BCTs than non-effective interventions. The most effective BCT was problem solving, a technique which encourages participants to generate potential strategies for health behaviour change (such as overcoming barriers, relapse prevention, and coping planning), and then selecting, applying, and evaluating the most appropriate strategy.^21,23,68^ Such strategies may have empowered participants to build the necessary skills to maintain healthier behaviours long term and prevent or overcome weight loss plateaus.

Collectively, the evidence suggests that technology-driven DPIs containing a larger number of BCTs were more likely to achieve clinically significant weight loss. Moreover, a specific set of seven BCTs were frequently identified in interventions that were effective in both the short and long term. Social support and adding objects to the environment (via pedometer use) were the most effective BCTs in the short term, and problem solving was the most effective BCT in the long term.

### Digital features

Much like the evidence for BCTs, interventions which used a larger number of passive and interactive digital features were more effective. Comparable results were reported by Donevant et al.^24^ and Holcombe et al.,^25^ who found that mobile health interventions were more effective in improving diabetes-related outcomes when interactive features were included. However, in the present review, the influence of interactive features decreased over time. Three digital features, all passive (health and lifestyle information and advice, diet tracking, and activity tracking) were frequently identified in effective interventions – suggesting that these components may constitute an effective core set of features which future technology-driven DPIs should integrate as a base standard.

In the short term, effective interventions used, on average, 1.9 more passive features and one more interactive feature than non-effective interventions. The most effective were the passive features of activity tracking and diet tracking and the interactive feature of online health coaching. In the long term, effective interventions used, on average, 1.9 more passive features and 0.25 more interactive features than non-effective interventions. The most effective were the passive features of: activity tracking, reminders and prompts, weight and biomeasure tracking, and the interactive feature of online health coaching.

The comparatively high use of digital tracking and online health coaching across effective interventions at both time periods offers two conclusions. First, self-monitoring may be most effective when digital technologies are used to track behaviours and outcomes. This is further supported by the low rate of effectiveness among interventions that used paper-based tracking only. Paper-based diaries can be burdensome and subject to delayed reporting and low adherence^69,70^ – limitations previously observed in diet plus physical activity interventions.^71,72^ However, paper-based reporting may have simply been less engaging for participants who chose to enrol in a technology-driven DPI through an interest in using digital tools. Second, feedback was most effective when delivered digitally, provided that it was given by a human coach. Online coaching predominantly involved two-way instant messaging and may have multiple advantages over automated feedback and real-time health coaching delivered in person or by phone. Online coaching grants participants the human interaction and detailed, tailored feedback that is lacking in automated feedback protocols; yet, unlike live coaching, instant messages are concise and accessible 24 hours a day. Furthermore, online coaching eliminates the need to set mutually convenient meeting times, arrange transport, or seek privacy to accept or make a phone call. Although self-monitoring and health coaching were most effective when delivered digitally, the same was not found for social support. The social support (unspecified) BCT, used in 100% of effective interventions, captured online, face-to-face, and phone support; yet the digitally-exclusive social media and support feature was found in only 50% of effective interventions, together suggesting that online support (e.g., via other participants) and face-to-face or phone support (e.g., via family, friends, and support staff) were equally effective.

Technology-driven DPIs have been developed to overcome the accessibility barriers of face-to-face interventions, and, as the present findings collectively suggest, interventions which use more BCTs and digital features are more effective; websites and smartphones may be the most suitable modes of delivery due to their increasingly high adoption rates and breadth of functionality. In 2018, internet use and smartphone ownership rates among adults in advanced economies were 90% and 76% respectively, with sharp, steady growth reported among the 50-and-older age group.^73^ Moreover these multimedia platforms have the capacity to incorporate a large variety of passive and interactive features and deliver a comprehensive, evidence-based curriculum such as that used in the Diabetes Prevention Program.

### Strengths and limitations

This was the first review of technology-driven DPIs to identify the BCTs and digital features frequently associated with clinically significant weight loss. We used two separate approaches to identify intervention components, enabling a detailed assessment of the interventions’ active ingredients. BCT coding represented a top-down approach in which intervention descriptions were reduced to their smallest behaviour change components as informed by existing labels and definitions. Conversely, digital feature identification was a bottom-up approach through which the features were informed by the intervention descriptions themselves – working from the narrowly defined digital components, up to the broadly defined digital features. Future reviews of interventions containing both digital and non-digital components may benefit from this dual-approach as, in addition to identifying the interventions’ most effective behavioural components, this approach can also identify a component’s most effective mode of delivery.

This review has some limitations. First, identification of BCTs and digital components was dependent on the detail in which the interventions were described – a common limitation of reviews that examine BCTs and digital features.^20,24,57^ While the imputation process mitigated this to some degree, imputation was only used to extrapolate BCTs from other studies within this review that applied the same standardised intervention. For all independent interventions, a BCT was only marked as present if its inclusion was explicitly clear in the intervention description(s). Second, although this review found BCTs and digital features to be identified more frequently in effective interventions, the long term assessment contained fewer interventions than the short term assessment. Therefore, greater confidence may be placed in the short term findings. Third, as a meta-analysis was not feasible (nor an aim of this review), an overall intervention effect was not established, and, as there was wide heterogeneity in sample size between studies, an intervention’s effectiveness may have been influenced by each study’s statistical power. However, to establish the effectiveness of individual interventions, we used international benchmarks and certification criteria that are applied, in practice, to assess interventions on a case-by-case basis. For example, the CDC can certify an individual cite regardless of sample size (at a minimum of 5 participants), provided the 5% weight loss benchmark was achieved.^8^ Finally, we reviewed studies with varying designs, including RCTs and non-experimental (observational) studies, which may have introduced various biases. However, technology-driven DPIs are designed for real world implementation, and RCT conditions are unlikely to match those in which the intervention is routinely completed. Furthermore, observational data can offer insight into the outcomes of participants often unrepresented in RCTs, such as older adults or individuals with comorbid conditions.^74^ These population groups are particularly important, with recent US reports citing that nearly half of adults aged 65 and over have prediabetes and, of all adults with prediabetes, rates of comorbid hypertension and dyslipidaemia were 51% and 24% respectively.^75,76^

### Future directions

Although this review described the associations between specific BCTs, digital features, and effectiveness, causality cannot be inferred, and further research is needed to determine the most effective intervention components for population sub-groups such as those defined by age, gender, ethnicity, geographic location, and socio-economic status. As some technology-driven DPIs have standardisation requirements, precluding the post-hoc testing of individual components,^35,77^ developers of future interventions could trial individual components during the development phase. For example, the Multiphase Optimisation Strategy (MOST),^78^ which facilitates the identification and testing of candidate components before a complete prototype is developed and ultimately tested via RCT. However, this process is subject to relatively high resource and time commitments, and care is needed to ensure that methodological rigour does not impede the assessment of real world effectiveness. Further research is also needed to identify the implementation and sustainability costs for the digital features by mode of delivery so that cost-effectiveness can be established. The ‘non-effective’ interventions in this review do not necessarily lack utility in T2D prevention as for every kilogram of weight lost in the original Diabetes Prevention Program, T2D risk was still reduced by 16%.^79^ Interventions that achieve modest weight loss but are inexpensive to sustain may still be viable T2D prevention tools. Finally, each of the reviewed interventions targeted physical activity and dietary behaviours, yet only 11 and 9 studies reported changes in these respective behaviours, each measured in a variety of ways. Standardised physical activity and dietary measures should be used in future interventions to enable researchers to identify the behaviours that most strongly influence weight loss. Additionally, as attrition varied widely between studies, further research is also required to assess participant adherence and engagement, and its subsequent impact on behaviour change and the outcomes associated with T2D.

## Conclusion

A number of technology-driven DPIs achieved clinically significant weight loss in adults at risk of developing T2D, particularly in the short term, which, along with reports of improved glycaemia and lower T2D incidence, supports the utility of these interventions for preventing diabetes. However, many interventions still fell short of reaching the 12 month 5% weight loss target as set by the CDC and recommended by NICE. Effective interventions contained a larger number of BCTs and digital features than non-effective interventions. Interventions that encouraged participants to set goals; self-monitor their diet, physical activity, and body weight; seek social support; and develop problem solving strategies were most successful. Technology-driven DPIs can be optimised by integrating digital-only tools that provide health and lifestyle information and advice; track behaviours and outcomes; and facilitate online behavioural support from a health coach. Websites and smartphone applications are appropriate modes of delivery as these multimedia platforms are widely accessible and have the capacity to incorporate a large variety of features. Additional research is needed to determine the cost-effectiveness of technology-driven DPIs and identify the mechanisms in which BCTs and digital features directly influence physical activity, dietary behaviours, and engagement among different population groups.

## Supplementary Material

Supplementary material

Supplementary material

Supplementary material

Supplementary material

Supplementary material

Supplementary material

Supplementary material
