# Supplementary material for: A systematic review of the behaviour change techniques and digital features in technology-driven type 2 diabetes prevention interventions
Source: Digit Health. 2020 Mar 24;6:2055207620914427. doi: 10.1177/2055207620914427 (PMC7093696; doi:10.1177/2055207620914427)
Supplement: Supplementary material [file DHJ914427_Supplementary_Material2.pdf]

Search Strategy

PubMed

1. Diabetes Mellitus, Type 2 [MeSH Term]
2. Prediabetic State [MeSH Term]
3. 1 OR 2
4. Preventive Health Services [MeSH Term]
5. Risk [MeSH Term]
6. Risk Reduction Behaviour [MeSH Term]
7. Education* [Text Word]
8. Intervention* [Text Word]
9. Prevention [Text Word]
10. Program* [Text Word]
11. OR 4/10
12. Cell Phone [MeSH Term]
13. Computers [MeSH Term]
14. Computers, Handheld [MeSH Term]
15. Electronic Mail [MeSH Term]
16. Fitness Trackers [MeSH Term]
17. Internet [MeSH Term]
18. Mobile Applications [MeSH Term]
19. Multimedia [MeSH Term]
20. Smartphone [MeSH]
21. Social Media [MeSH Term]
22. Software [MeSH Term]
23. Telemedicine [MeSH Term]
24. Telephone [MeSH Term]
25. Television [MeSH Term]
26. Text Messaging [MeSH Term]
27. Video Games [MeSH Term]
28. Video-Audio Media [Publication Type] [MeSH Term]
29. Virtual Reality [MeSH Term]
30. Wearable Electronic Device [MeSH Term]
31. Webcasts [Publication Type] [MeSH Term]
32. Digital [Text Word]
33. DVD* [Text Word]
34. Electronic [Text Word]
35. Online [Text Word]
36. Pedometer* [Text Word]
37. Sensor* [Text Word]
38. SMS [Text Word]
39. Technolog* [Text Word]
40. TV [Text Word]
41. OR 12/40
42. Adipose Tissue [MeSH Term]
43. Blood Glucose [MeSH Term]
44. Body Mass Index [MeSH Term]
45. Body Weight [MeSH Term]
46. Body Weight Changes [MeSH Term]
47. Glucose Intolerance [MeSH Term]

48. Glucose Tolerance Test [MeSH Term]
49. Glycated Hemoglobin A [MeSH Term]
50. Incidence [MeSH Term]
51. Prevalence [MeSH Term]
52. Waist Circumference [MeSH Term]
53. Waist-Hip Ratio [MeSH Term]
54. OR 42/53
55. 3 AND 11 AND 41 AND 54
56. Diabetes Mellitus, Type 1 [MeSH Term]
57. 55 NOT 56

CINAHL, EMBASE, MEDLINE, PsycINFO

1. Diabet*.ti.
2. Prediabet*.ti.
3. Pre-diabet*.ti.
4. OR 1/3
5. Education*.ti.
6. Intervention*.ti.
7. Prevent*.ti.
8. Program*.ti.
9. (risk adj2 reduc*).ti.
10. OR 5/9
11. “Social media”.tw.
12. App.tw.
13. Apps.tw.
14. Computer.tw.
15. Digital.tw.
16. DVD*.tw.
17. eHealth.tw.
18. e-Health.tw.
19. Electronic.tw.
20. eMail*.tw.
21. Internet.tw.
22. mHealth.tw.
23. m-Health.tw.
24. Mobile.tw.
25. Multimedia.tw.
26. Online.tw.
27. Pedometer*.tw.
28. Phone.tw.
29. Sensor*.tw.
30. Smartphone.tw.
31. SMS.tw.
32. Software.tw.
33. Technolog*.tw.
34. Telehealth.tw.
35. Telephone.tw.
36. Television.tw.

37. Text.tw.
38. Tracker*.tw.
39. TV.tw.
40. Video*.tw.
41. Virtual.tw.
42. Wearable*.tw.
43. Web*.tw.
44. OR 11/43
45. “Body fat”.tw.
46. “Body Mass Index”.tw.
47. “Fasting blood”.tw.
48. “Fasting glucose”.tw.
49. “Fasting plasma”.tw.
50. “Glucose tolerance”.tw.
51. “Waist circumference”.tw.
52. A1c.tw.
53. BMI.tw.
54. Glycated.tw.
55. HbA1c.tw.
56. Incidence.tw.
57. Prevalence.tw.
58. Waist-hip.tw.
59. Waist-to-hip.tw.
60. Weight.tw.
61. OR 45/61
62. 4 AND 10 AND 44 AND 61
63. “Type 1”.ti.
64. 62 NOT 63
