# Supplementary material for: A systematic review of the behaviour change techniques and digital features in technology-driven type 2 diabetes prevention interventions
Source: Digit Health. 2020 Mar 24;6:2055207620914427. doi: 10.1177/2055207620914427 (PMC7093696; doi:10.1177/2055207620914427)
Supplement: Supplementary material [file DHJ914427_Supplementary_Material3.pdf]

References for All Included Articles

Study (year)	Reference(s)
Aguiar et al. (2016)	*Aguiar, E. J., Morgan, P. J., Collins, C. E., Plotnikoff, R. C., Young, M. D., & Callister, R. (2016). Efficacy of the type 2 diabetes prevention using lifestyle education program RCT. *American Journal of Preventive Medicine, 50*(3), 353-364. doi: 10.1016/j.amepre.2015.08.020
	Aguiar, E. J., Morgan, P. J., Collins, C. E., Plotnikoff, R. C., Young, M. D., & Callister, R. (2014). The PULSE (Prevention Using LifeStyle Education) trial protocol: a randomised controlled trial of a Type 2 Diabetes Prevention programme for men. *Contemporary Clinical Trials*, *39*(1), 132-144. doi: 10.1016/j.cct.2014.07.008
	Aguiar, E. J., Morgan, P. J., Collins, C. E., Plotnikoff, R. C., Young, M. D., & Callister, R. (2017). Process evaluation of the type 2 diabetes mellitus PULSE program randomized controlled trial: recruitment, engagement, and overall satisfaction. *American Journal of Men's Health*, *11*(4), 1055-1068. doi: 10.1177/1557988317701783
	Rollo, M. E., Aguiar, E. J., Pursey, K. M., Morgan, P. J., Plotnikoff, R. C., Young, M. D., ... & Callister, R. (2017). Impact on dietary intake of a self-directed, gender-tailored diabetes prevention program in men. *World Journal of Diabetes, 8*(8), 414. doi: 10.4239/wjd.v8.i8.414
	Morgan, P. J., Callister, R., Collins, C. E., Plotnikoff, R. C., Young, M. D., Berry, N., ... & Saunders, K. L. (2012). The SHED-IT community trial: a randomized controlled trial of internet-and paper-based weight loss programs tailored for overweight and obese men. *Annals of Behavioral Medicine, 45*(2), 139-152. doi: 10.1007/s12160-012-9424-z
	Morgan, P. J., Collins, C. E., Plotnikoff, R. C., McElduff, P., Burrows, T., Warren, J. M., ... & Callister, R. (2010). The SHED-IT community trial study protocol: a randomised controlled trial of weight loss programs for overweight and obese men. *BMC Public Health, 10*(1), 701. doi: 10.1186/1471-2458-10-701
Arens et al. (2018)	*Arens, J. H., Hauth, W., & Weissmann, J. (2018). Novel app-and web-supported diabetes prevention program to promote weight reduction, physical activity, and a healthier lifestyle: observation of the clinical application. *Journal of Diabetes Science and Technology, 12*(4), 831-838. doi: 10.1177/1932296818768621

Study	Reference(s)
Block et al. (2015)	*Block, G., Azar, K. M., Romanelli, R. J., Block, T. J., Hopkins, D., Carpenter, H. A., ... & Block, C. H. (2015). Diabetes prevention and weight loss with a fully automated behavioral intervention by email, web, and mobile phone: a randomized controlled trial among persons with prediabetes. *Journal of Medical Internet Research, 17(*10), e240. doi: 10.2196/jmir.4897
	Block, G., Azar, K. M., Block, T. J., Romanelli, R. J., Carpenter, H., Hopkins, D., ... & Block, C. H. (2015). A fully automated diabetes prevention program, Alive-PD: program design and randomized controlled trial protocol. *JMIR Research Protocols, 4*(1), e3. doi: 10.2196/resprot.4046
	Block, G., Azar, K. M. J., Romanelli, R. J., Block, T. J., Palaniappan, L. P., Dolginsky, M., & Block, C. H. (2016). Improving diet, activity and wellness in adults at risk of diabetes: randomized controlled trial. *Nutrition & Diabetes, 6*(9), e231. doi: 10.1038/nutd.2016.42
Castro Sweet et al. (2018)	*Castro Sweet, C. M., Chiguluri, V., Gumpina, R., Abbott, P., Madero, E. N., Payne, M., ... & Prewitt, T. (2018). Outcomes of a digital health program with human coaching for diabetes risk reduction in a Medicare population. *Journal of Aging and Health, 30(*5), 692-710. doi: 10.1177/0898264316688791
Cha et al. (2014)	*Cha, E., Kim, K. H., Umpierrez, G., Dawkins, C. R., Bello, M. K., Lerner, H. M., ... & Dunbar, S. B. (2014). A feasibility study to develop a diabetes prevention program for young adults with prediabetes by using digital platforms and a handheld device. *The Diabetes Educator, 40*(5), 626-637. doi: 10.1177/0145721714539736
	Cha, E., Umpierrez, G., Kim, K. H., Bello, M. K., & Dunbar, S. B. (2013). Characteristics of American young adults with increased risk for type 2 diabetes: a pilot study. *The Diabetes Educator, 39*(4), 454-463. doi: 10.1177/0145721713486199
Estabrooks and Smith-Ray (2008)	*Estabrooks, P. A., & Smith-Ray, R. L. (2008). Piloting a behavioral intervention delivered through interactive voice response telephone messages to promote weight loss in a pre-diabetic population. *Patient Education and Counseling, 72*(1), 34-41. doi: 10.1016/j.pec.2008.01.007
Everett et al. (2018)	*Everett, E., Kane, B., Yoo, A., Dobs, A., & Mathioudakis, N. (2018). A novel approach for fully automated, personalized health coaching for adults with prediabetes: pilot clinical trial. *Journal of Medical Internet Research,* 20(2), e72. doi: 10.2196/jmir.9723

Study	Reference(s)
Fischer et al. (2016)	*Fischer, H. H., Fischer, I. P., Pereira, R. I., Furniss, A. L., Rozwadowski, J. M., Moore, S. L., ... & Havranek, E. P. (2016). Text message support for weight loss in patients with prediabetes: a randomized clinical trial. *Diabetes Care, 39*(8), 1364-1370. doi: 10.2337/dc15-2137
Fukuoka et al. (2015)	*Fukuoka, Y., Gay, C. L., Joiner, K. L., & Vittinghoff, E. (2015). A novel diabetes prevention intervention using a mobile app: a randomized controlled trial with overweight adults at risk. *American Journal of Preventive Medicine, 49*(2), 223-237. doi: 10.1016/j.amepre.2015.01.003
Kramer et al. (2010)	*Kramer, M. K., Kriska, A. M., Venditti, E. M., Semler, L. N., Miller, R. G., McDonald, T., ... & Orchard, T. J. (2010). A novel approach to diabetes prevention: evaluation of the Group Lifestyle Balance program delivered via DVD. *Diabetes Research and Clinical Practice, 90*(3), e60-e63. doi: 10.1016/j.diabres.2010.08.013
Limaye et al. (2017)	*Limaye, T., Kumaran, K., Joglekar, C., Bhat, D., Kulkarni, R., Nanivadekar, A., & Yajnik, C. (2017). Efficacy of a virtual assistance-based lifestyle intervention in reducing risk factors for Type 2 diabetes in young employees in the information technology industry in India: LIMIT, a randomized controlled trial. *Diabetic Medicine, 34*(4), 563-568. doi: 10.1111/dme.13258
Ma et al. (2013)	*Ma, J., Yank, V., Xiao, L., Lavori, P. W., Wilson, S. R., Rosas, L. G., & Stafford, R. S. (2013). Translating the Diabetes Prevention Program lifestyle intervention for weight loss into primary care: a randomized trial. *JAMA Internal Medicine, 173*(2), 113-121. doi: 10.1001/2013.jamainternmed.987<br>Azar, K. M., Xiao, L., & Ma, J. (2013). Baseline obesity status modifies effectiveness of adapted diabetes prevention program lifestyle interventions for weight management in primary care. *BioMed Research International*, 2013. doi: 10.1155/2013/191209<br>Ma, J., King, A. C., Wilson, S. R., Xiao, L., & Stafford, R. S. (2009). Evaluation of lifestyle interventions to treat elevated cardiometabolic risk in primary care (E-LITE): a randomized controlled trial. *BMC Family Practice, 10*(1), 71. doi: 10.1186/1471-2296-10-71<br>Ma, J., Xiao, L., & Blonstein, A. C. (2013). Measurement of self-monitoring web technology acceptance and use in an e-health weight-loss trial. *Telemedicine and e-Health, 19*(10), 739-745. doi: 10.1089/tmj.2013.0009<br>Xiao, L., Yank, V., Wilson, S. R., Lavori, P. W., & Ma, J. (2013). Two-year weight-loss maintenance in primary care-based Diabetes Prevention Program lifestyle interventions. *Nutrition & Diabetes, 3*(6), e76. doi: 10.1038/nutd.2013.17

Study	Reference(s)
Michaelides et al. (2016)	*Michaelides, A., Raby, C., Wood, M., Farr, K., & Toro-Ramos, T. (2016). Weight loss efficacy of a novel mobile Diabetes Prevention Program delivery platform with human coaching. *BMJ Open Diabetes Research and Care, 4*(1), e000264. doi: 10.1136/bmjdrc-2016-000264<br>Michaelides, A., Major, J., Pienkosz Jr, E., Wood, M., Kim, Y., & Toro-Ramos, T. (2018). Usefulness of a novel mobile diabetes prevention program delivery platform with human coaching: 65-week observational follow-up. *JMIR mHealth and uHealth, 6*(5), e93. doi: 10.2196/mhealth.9161
Piatt et al. (2013)	*Piatt, G. A., Seidel, M. C., Powell, R. O., & Zgibor, J. C. (2013). Comparative effectiveness of lifestyle intervention efforts in the community: results of the Rethinking Eating and ACTivity (REACT) study. *Diabetes Care, 36*(2), 202-209. doi: 10.2337/dc12-0824<br>Piatt, G. A., Seidel, M. C., Powell, R. O., & Zgibor, J. C. (2016). Influence of patient-centered decision making on sustained weight loss and risk reduction following lifestyle intervention efforts in rural Pennsylvania. *The Diabetes Educator, 42*(3), 281-290. doi: 10.1177/0145721716636962
Ramachandran et al. (2013)	*Ramachandran, A., Snehalatha, C., Ram, J., Selvam, S., Simon, M., Nanditha, A., ... & Oliver, N. (2013). Effectiveness of mobile phone messaging in prevention of type 2 diabetes by lifestyle modification in men in India: a prospective, parallel-group, randomised controlled trial. *The Lancet Diabetes & Endocrinology, 1(*3), 191-198. doi: 10.1016/S2213-8587(13)70067-6
Sepah et al. (2014)	*Sepah, S. C., Jiang, L., & Peters, A. L. (2014). Translating the diabetes prevention program into an online social network: validation against CDC standards. *The Diabetes Educator, 40*(4), 435-443. doi: 10.1177/0145721714531339<br>Sepah, S. C., Jiang, L., Ellis, R. J., McDermott, K., & Peters, A. L. (2017). Engagement and outcomes in a digital Diabetes Prevention Program: 3-year update. *BMJ Open Diabetes Research and Care, 5*(1), e000422. doi: 10.1136/bmjdrc-2017-000422<br>Sepah, S. C., Jiang, L., & Peters, A. L. (2015). Long-term outcomes of a Web-based diabetes prevention program: 2-year results of a single-arm longitudinal study. *Journal of Medical Internet Research, 17*(4), e92. doi: 10.2196/jmir.4052<br>Su, W., Chen, F., Dall, T. M., Iacobucci, W., & Perreault, L. (2016). Return on Investment for Digital Behavioral Counseling in Patients With Prediabetes and Cardiovascular Disease. *Preventing Chronic Disease, 13*, E13. doi:10.5888/pcd13.150357

Study	Reference(s)
Tate et al. (2003)	*Tate, D. F., Jackvony, E. H., & Wing, R. R. (2003). Effects of Internet behavioral counseling on weight loss in adults at risk for type 2 diabetes: a randomized trial. *JAMA, 289*(14), 1833-1836. doi: 10.1001/jama.289.14.1833
Wong et al. (2013)	* Wong, C. K., Fung, C. S., Siu, S. C., Lo, Y. Y., Wong, K. W., Fong, D. Y., & Lam, C. L. (2013). A short message service (SMS) intervention to prevent diabetes in Chinese professional drivers with pre-diabetes: a pilot single-blinded randomized controlled trial. *Diabetes Research and Clinical Practice, 102*(3), 158-166. doi: 10.1016/j.diabres.2013.10.002
	Wong, C. K., Jiao, F. F., Siu, S. C., Fung, C. S., Fong, D. Y., Wong, K. W., ... & Lam, C. L. (2016). Cost-effectiveness of a short message service intervention to prevent type 2 diabetes from impaired glucose tolerance. *Journal of Diabetes Research, 2016*. doi: 10.1155/2016/1219581
Wilson et al. (2017)	* Wilson, M. G., Sweet, C. M. C., Edge, M. D., Madero, E. N., McGuire, M., Pilsmaker, M., ... & Kirschner, S. (2017). Evaluation of a digital behavioral counseling program for reducing risk factors for chronic disease in a workforce. *Journal of Occupational and Environmental Medicine, 59*(8), e150. doi: 10.1097/JOM.0000000000001091

*Main study paper
