# Supplementary material for: A systematic review of the behaviour change techniques and digital features in technology-driven type 2 diabetes prevention interventions
Source: Digit Health. 2020 Mar 24;6:2055207620914427. doi: 10.1177/2055207620914427 (PMC7093696; doi:10.1177/2055207620914427)
Supplement: Supplementary material [file DHJ914427_Supplementary_Material4.pdf]

Supplementary Table 1.

Quality Assessment Summaries for All Studies

Checklist criteria		Aguiar (2016)	Arens (2018)	Block (2015)	Castro Sweet (2017)	Cha (2014)	Estabrooks (2008)	Everett (2018)	Fischer (2016)	Fukuoka (2015)	Kramer (2010)	Limaye (2017)	Ma (2013)	Michaelides (2016)	Piatt (2013)	Ramachandran (2013)	Sepah (2014)	Tate (2003)	Wilson (2017)	Wong (2013)
1.1	Source population or area well described	++	+	++	+	++	++	++	++	++	++	++	++	+	++	++	+	++	++	++
1.2	Eligible population or area representative	++	+	++	+	++	+	+	++	++	++	++	+	+	++	++	+	+	++	++
1.3	Selected participants or areas representative	++	+	++	++	+	++	++	++	+	+	+	+	+	++	++	+	+	++	++
2.1	Allocation: selection bias minimised	++	+	++	NA	NA	++	+	++	++	+	++	++	NA	+	++	NA	++	NA	++
2.2	Interventions (& comparisons) well described & appropriate	++	+	++	++	++	++	++	++	++	++	++	++	+	++	++	++	++	++	++
2.3	Allocation concealed	++	NR	NR	NA	NA	NR	+	NR	++	NA	NR	NR	NA	+	+	NA	NR	NA	+
2.4	Participants &/or investigators blinded	NA	NA	NA	NA	NA	NA	NA	NA	NA	NA	NA	NA	NA	NA	NA	NA	NA	NA	NA
2.5	Exposure to intervention & comparison adequate	++	+	++	NA	NA	++	++	++	++	++	++	++	NA	+	++	NA	++	NA	++
2.6	Contamination Acceptably low	++	++	++	NA	NA	++	++	+	++	++	++	++	NA	++	++	NA	++	NA	++
2.7	Other interventions similar in groups	++	++	++	NA	NA	++	+	+	++	++	++	+	NA	+	++	NA	++	+	++
2.8	All participants accounted for at study conclusion	++	+	++	++	++	+	++	++	++	+	+	++	++	+	++	+	++	+	+

2.9	Setting reflects usual UK practice	+	++	++	+	+	++	+	++	++	++	+	++	+	+	+	+	+	+	+
2.10	Intervention or control reflects usual UK practice	+	++	++	+	+	++	+	++	++	++	+	++	+	+	+	+	++	+	+
3.1	Outcome measures reliable	++	+	++	++	++	++	++	++	++	++	++	++	++	++	++	++	++	++	++
3.2	Outcome measures complete	++	++	++	+	++	++	++	++	++	++	++	++	++	++	++	+	++	+	+
3.3	All important outcomes assessed	++	+	++	++	++	++	++	++	++	++	++	++	+	++	+	++	++	++	++
3.4	Outcomes relevant	++	++	++	++	++	++	++	++	++	++	++	++	++	++	++	++	++	++	++
3.5	Similar follow-up times in groups	++	+	++	NA	NA	++	++	++	++	++	++	++	NA	++	++	NA	++	NA	++
3.6	Follow-up time meaningful	+	+	+	++	+	+	+	++	+	+	++	++	+	++	++	++	++	++	++
4.1	Groups similar at baseline	++	+	++	NA	NA	++	+	++	++	+	++	++	NA	++	++	NA	++	NA	++
4.2	ITT analysis conducted	++	+	++	++	+	++	+	++	++	++	++	++	++	++	++	++	++	++	++
4.3	Study sufficiently powered	++	+	+	NR	+	NR	+	++	++	++	++	++	++	++	++	NR	+	NR	NR
4.4	Estimates of effect size given or calculable	++	++	+	++	++	++	++	+	++	++	++	++	++	+	++	++	++	++	++
4.5	Analytical methods appropriate	++	+	++	++	++	+	++	+	++	++	++	++	++	++	++	+	++	++	++
4.6	Precision of intervention effects given or calculable	++	++	++	++	++	++	++	++	++	++	++	++	++	++	++	++	++	++	++
5.1	Study results internally valid (i.e. unbiased)	++	+	++	++	+	++	+	++	++	++	++	++	++	+	++	++	++	++	++
5.2	Findings generalisable to source population (i.e. externally valid)	++	+	++	+	+	++	+	++	+	++	++	+	+	++	++	+	+	++	++

Supplementary Table 2.

The Proportion of Baseline Weight Lost at Each Follow-Up for Interventions Included in the Primary Effectiveness Analysis

Study (year)<br>*Intervention*	Follow-up point									
	Short Term				Long Term					
	3 months	16 weeks	5 months	6 months	12 months	15 months	65 weeks	18 months	24 months	36 months
Aguiar (2016)	**-3.74%**	-	-	**-4.85%**	-	-	-	-	-	-
Block (2018)	-	-	-	**-3.60%**	-	-	-	-	-	-
Castro Sweet (2017)	-	**-6.5%**	-	**-8.0%**	**-7.5%**	-	-	-	-	-
Cha (2014)*	-2.50%	-	-	-	-	-	-	-	-	-
Estabrooks (2008)	-2.63%	-	-	-	-	-	-	-	-	-
Everett (2018)*	-1.9%	-	-	-	-	-	-	-	-	-
Fischer (2016)		-	-	-1.93%	-1.35%	-	-	-	-	-
Fukuoka (2015)	**-5.8%**	-	**-6.8%**	-	-	-	-	-	-	-
Kramer (2010)	**-5.6%**	-	-	-	-	-	-	-	-	-
Limaye (2017)	-0.73%	-	-	-1.48%	-1.35%	-	-	-	-	-
Ma (2013)	**-4.9%**	-	-	**-4.7%**	-	**-5.0%**	-	-	-	-
Michaelides (2016)	-	**-5.65% (S)**<br>**-6.33% (C)**	-	**-6.58% (S)**<br>**-7.50% (C)**	-	-	**-6.15% (S)**<br>**-7.36% (C)**	-	-	-
Piatt (2013) *DVD*	**-5.67%**		-	**-3.49%**	-	-	-	-4.6%	-	-
Piatt (2013) *Internet*	**-6.26%**		-	**-3.11%**	-	-	-	**-5.25%**	-	-
Sepah (2014)	-	**-5.0% (S)**<br>**-5.2% (C)**	-	-	-4.7% (S)<br>-4.9% (C)	-	-	-	-4.2% (S)<br>-4.3% (C)	-3.0% (S)<br>-2.9% (C)
Tate (2003) *BI*	**-3.02%**	-	-	-2.80%	-2.24%	-	-	-	-	-
Tate (2003) *BeC*	**-4.76%**	-	-	**-6.03%**	-4.8%	-	-	-	-	-
Wilson (2017)	-	**-4.6%**	-	-	-0.93%	-	-	-	-	-
Wong (2013)	-	-	-	-0.69%	-1.57%	-	-	-	-1.47%	-

Note: S: starters, defined as participants who read at least one article during any 4 of the 16 initial intervention weeks; C: Completers, defined as participants who read at least one article per week during any 9 of the 16 weeks. Bold text denotes clinically significant weight loss.

*Studies that applied per-protocol or similar analyses only, rather than intention-to-treat.

Supplementary Table 3.

Changes in Body Weight and Glycaemia from Baseline to Most Recent Follow-up (Expressed in Mean Values Unless Otherwise Indicated)

Author(s)<br>(year)<br>*Intervention*	Weight change	Proportion of sample that achieved target weight loss[‡]	Change in A1c	Change in Fasting Glucose
Aguiar et al.<br>(2016)	*At 6 months*<br>Intervention (n = 53)<br>-5 kg (p < .05)*<br>Control (n = 48)<br>+0.5 kg (p > .05)<br>[p < .001, d = 1.15]*	*At 6 months*<br>5% weight loss<br>Intervention: 42.1%<br>Control: 4.8%<br>[p < .001]*	*At 6 months*<br>Intervention (n = 53)<br>-0.4% (p < .05)*<br>Control (n = 48)<br>-0.2% (p < .05)*<br>[p = .002, d = 0.64]*	*FPG at 6 months*<br>Intervention (n = 53)<br>-0.08 mmol/L (p > .05)<br>Control (n = 48)<br>-0.03 mmol/L (p > .05)<br>[p = .742, d = 0.07]
Arens et al.<br>(2018) †	*Intervention: At 8.3 months*<br>*Standard care: at 11.6 months*<br>Intervention (n = 109)<br>-2.4 kg (p < .0001)*<br>Standard Care (n = 57)<br>-0.01kg (p = .99)<br>[p = .057] adj. for baseline	*Over time*<br>Chance to achieve 5% weight reduction.<br>Intervention: 6.2 times greater than Standard Care	Not Reported	Not Reported
Block et al.<br>(2015)	*At 6 months*<br>Intervention (n = 163)<br>-3.3 kg<br>Control (n = 176)<br>-1.26 kg<br>[p < .001]*	*At 6 months*<br>5% weight loss<br>Intervention: 35.3%<br>Control: 8.3%	*At 6 months*<br>Intervention (n = 163)<br>-0.26%<br>Control (n = 176)<br>-0.18%<br>[p < .001]*	*FG at 6 months*<br>Intervention (n = 163)<br>-0.41 mmol/L<br>Control (n = 176)<br>-0.12 mmol/L<br>[p < .001]*
Castro Sweet et al.<br>(2018)	*At 12 months*<br>Intervention (n = 501)<br>-7.1 kg (p = .001)*<br>Control: NA	Not Reported	*At 12 months*<br>Intervention (n = 69)<br>-0.14% (p = .0001)*<br>Control: NA	Not Reported

Author(s)<br>(year)<br>*Intervention*	Weight change	Proportion of sample that achieved target weight loss‡	Change in A1c	Change in Fasting Glucose
Cha et al.<br>(2014) †	*At 3 months*<br>Intervention (*n* = 13)<br>-2.9 kg<br>(*p* = .031, *d* = -0.12)*<br>Control: NA	Not<br>Reported	*At 3 months*<br>Intervention (*n* = 13)<br>-0.4%<br>(*p* = .007, *d* = -0.76)*<br>Control: NA	*FG at 3 months*<br>Intervention (*n* = 13)<br>+0.28 mmol/L (*p* = .112, *d* = 0.39)<br>Control: NA
Estabrooks and Smith-Ray<br>(2008)	*At 3 months*<br>Intervention (*n* = 28)<br>-2.3 kg<br>Control (*n* = 31)<br>-2 kg<br>[*p* = .13] [when adjusting for baseline values]	Not<br>Reported	Not<br>Reported	Not<br>Reported
Everett et al.<br>(2018) †	*At 3 months*<br>Intervention (*n* = 38)<br>-1.6 kg (*p* = .02)*<br>Calibration (*n* = 9): not reported	Not<br>Reported	*At 3 months*<br>*Change in median values*<br>Intervention (*n* = 38)<br>-0.10% (*p* = .04)*<br>Calibration (*n* = 9): not reported	*FG at 3 months*<br>*Change in median values*<br>Intervention (*n* = 38)<br>-0.01 mmol/L (*p* = .59)<br>Calibration (*n* = 9): not reported
Fischer et al.<br>(2016)	*At 12 months*<br>Intervention (*n* = 78)<br>-2.6 lbs<br>Control (*n* = 79)<br>-0.56 lbs<br>[*p* = .05]	*At 12 months*<br>5% weight loss<br>Intervention: 38.5%<br>Control: 21.5%<br>[*p* = .02]*	*At 12 months*<br>Intervention (*n* = 78)<br>-0.09%<br>Control (*n* = 79)<br>+0.19%<br>[*p* = .07]	Not<br>Reported
Fukuoka et al.<br>(2015)	*At 5 months*<br>Intervention (*n* = 30)<br>-6.2 kg<br>Control (*n* = 31)<br>+0.3 kg<br>[*p* < .001]*	*At 5 months*<br>10% weight loss<br>Intervention: 29%<br>Control: 0%	*At 5 months*<br>Intervention (*n* = 30)<br>-0.10%<br>Control (*n* = 31)<br>-0.04%<br>[*p* = .25]	*FPG at 5 months*<br>Intervention (*n* = 30)<br>-0.02 mmol/L<br>Control (*n* = 31)<br>+0.02 mmol/L<br>[*p* = .63]

Author(s) (year) *Intervention*	Weight change	Proportion of sample that achieved target weight loss[‡]	Change in A1c	Change in Fasting Glucose
Kramer et al. (2010)	*At 3 months*<br>DVD (n = 22)<br>-5.4 kg (p < .0001)*<br>Face-to-face (n = 26)<br>-6.3 kg (p < .0001)*	Not Reported	*At 3 months*<br>DVD (n = 21)<br>-0.16% (p = .002)*<br>Face-to-face (n = 26)<br>-0.31% (p < .0001)*	*FG at 3 months*<br>DVD (n = 21)<br>-0.26 mmol/L (p = .003)*<br>Face-to-face (n = 26)<br>+0.06 mmol/L (p = .098)
Limaye et al. (2017)	*At 12 months*<br>Intervention (n = 133)<br>-1 kg<br>Control (n = 132)<br>+0.7 kg<br>[p < .001]*	Not Reported	Not Reported	*FPG at 12 months*<br>Intervention (n = 133)<br>+0.19 mmol/L<br>Control (n = 132)<br>+0.33 mmol/L<br>[p = .022]*
Ma et al. (2013)	*At 15 months*<br>Self-directed (n = 81)<br>-4.5 kg<br>Coach-led (n = 79)<br>-6.3 kg<br>Usual Care (n = 81)<br>-2.4.kg<br>Self-directed vs Usual Care<br>[p = .02]*	*At 15 months*<br>5%, 7%, and 10% weight loss<br>Intervention: 46.9%, 37.6%, and 17.2%<br>Usual care: 24.6%, 14.7%, and 3.5%<br>[p = .007*, p = .006*, and p = .01*]	Not Reported	*FPG at 15 months*<br>Self-directed (n = 81)<br>-0.15 mmol/L<br>Coach-led (n = 79)<br>-0.23 mmol/L<br>Usual Care (n = 81)<br>+0.01 mmol/L<br>Self-directed vs Usual Care<br>[p = .01]*
Michaelides et al. (2016)	*At 65 weeks*<br>Starters (n = 59)<br>-5.9 kg (p < .001)*<br>Completers (n = 47)<br>-7.1 kg (p < .001)*	Not Reported	Not Reported	Not Reported

Author(s)<br>(year)<br>*Intervention*	Weight change	Proportion of sample that achieved target weight loss[‡]	Change in A1c	Change in Fasting Glucose
Piatt et al.<br>(2013)<br><br>*GLB-DVD*	*At 18 months*<br>DVD (n = 64)<br>-4.5 kg (p < .0001)*<br>Internet (n = 44)<br>-5.2 kg (p < .0001)*<br>Face-to-face (n = 96)<br>-4.9 kg (p < .0001)*<br>Self-selected (n = 56)<br>-5.9 kg (p < .0001)*	*At 6 months*<br>5% weight loss<br>DVD: 51.5%<br>Internet: 57.1%<br>F2F: 51.9%<br>SS: 66.7%	Not<br>Reported	Not<br>Reported
Piatt et al.<br>(2013)<br><br>*GLB-Internet*	*At 18 months*<br>Internet (n = 44)<br>-5.2 kg (p < .0001)*<br>DVD (n = 64)<br>-4.5 kg (p < .0001)*<br>Face-to-face (n = 96)<br>-4.9 kg (p < .0001)*<br>Self-selected (n = 56)<br>-5.9 kg (p < .0001)*	*At 6 months*<br>5% weight loss<br>Internet: 57.1%<br>DVD: 51.5%<br>F2F: 51.9%<br>SS: 66.7%	Not<br>Reported	Not<br>Reported
Ramachandran et al.<br>(2013)	Not<br>Reported	Not<br>Reported	Not<br>Reported	*FPG at 5 years*<br>Intervention (n = 171)<br>+0.4 mmol/L<br>Control (n = 157)<br>+0.2 mmol/L<br>[*not significant*]
Sepah et al.<br>(2014)	*At 3 years*<br>Core (n = 187)<br>-3 kg (p = .0009)*<br>Post-core (n = 144)<br>-2.9 kg (p = .0024)*	*At 12 months*<br>5% weight loss<br>Core: not reported<br>Post-core: 47%	*At 3 years*<br>Core (n = 187)<br>-0.31% (p = .0008)*<br>Post-core (n = 155)<br>-0.33% (p = .0005)*	Not<br>Reported

Author(s)<br>(year)<br>*Intervention*	Weight change	Proportion of sample that achieved target weight loss‡	Change in A1c	Change in Fasting Glucose
Tate et al.<br>(2003)<br><br>*Basic Internet*	*At 12 months*<br>Basic Int (*n* = 46)<br>-2 kg<br>Int + BeC (*n* = 46)<br>-4.4 kg<br>[*p* =.04]* favours BeC	Not Reported	Not Reported	*FBG at 12 months*<br>Basic Int (*n* = 46): not reported<br>Int + BeC (*n* = 46): not reported<br>[*p* = .93]
Tate et al.<br>(2003)<br><br>*Internet and Behavioural e-Counseling*	*At 12 months*<br>Int + BeC (*n* = 46)<br>-4.4 kg<br>Basic Int (*n* = 46)<br>-2 kg<br>[*p* =.04]* favours BeC	Not Reported	Not Reported	*FBG at 12 months*<br>Int + BeC (*n* = 46): NR<br>Basic Int (*n* = 46): NR<br>[*p* = .93]
Wilson et al.<br>(2017)	*At 12 months*<br>Intervention (*n* = 634)<br>-0.9 kg<br>Control (*n* = 1,268)<br>+0.6 kg<br>[*p* < .05]*	*At 12 months*<br>5% weight loss<br>Intervention: 31%<br>Control: 20%<br>[*p* < .001]*	Not Reported	*FBG at 12 months*<br>Intervention (*n* = 634)<br>-0.08 mmol/L<br>Control (1,268)<br>+0.01 mmol/L<br>[*p* < .05]*
Wong et al.<br>(2013)	*At 24 months*<br>Intervention (*n* = 54)<br>-1 kg<br>Control (*n* = 50)<br>-0.4 kg<br>[*p* = .094]	Not Reported	Not Reported	*FPG at 24 months*<br>Intervention (*n* = 54)<br>+0.03 mmol/L<br>Control (N = 50)<br>+0.04 mmol/L<br>[*p* = .468]

Note: For the purpose of standardisation, all body weights reported in lbs were converted to kg, and all fasting glucose measures reported in mg/dL were converted to mmol/L; (*p*): within-group result; [*p*]: between-group result; FG: fasting glucose; FBG: fasting blood glucose; FPG: fasting plasma glucose; NA: not applicable. *Statistically significant at $p < .05$; †studies that applied per-protocol or similar analyses rather than intention to treat; ‡weight loss target as described by the authors of each study.

Supplementary Table 4.

Behaviour Change Techniques Identified in All Interventions

No.	Behaviour Change Technique	Aguiar (2016)	Arens (2018)	Block (2015)	Castro Sweet (2017)	Cha (2014)	Estabrooks (2008)	Everett (2018)	Fischer (2016)	Fukuoka (2015)	Kramer (2010)	Limaye (2017)	Ma (2013)	Michaelides (2016)	Piatt (2013) *DVD*	Piatt (2013) *Internet*	Ramachandran (2013)	Sepah (2014)	Tate (2003) *BI*	Tate (2003) *BeC*	Wilson (2017)	Wong (2013)	TOTAL (Inc. Imp.)	TOTAL (Exc. Imp.)
Cluster One: Goals and planning																								
1.1	Goal setting (behaviour)	✓	✓	✓	*	✓	-	✓	*	*	*	✓	✓	*	*	*	-	✓	-	-	*	-	16	8
1.2	Problem solving	-	✓	-	*	✓	✓	-	✓	✓	*	-	✓	*	✓	✓	✓	*	-	-	*	-	14	9
1.3	Goal setting (outcome)	✓	✓	✓	*	-	-	✓	*	*	*	✓	✓	*	✓	✓	-	✓	-	-	*	-	15	9
1.4	Action planning	✓	-	✓	-	-	✓	-	-	-	*	-	✓	-	*	*	-	-	-	-	-	-	7	4
1.5	Review behaviour goals	-	-	-	-	✓	-	-	-	-	✓	-	✓	-	*	*	-	-	-	-	-	-	5	3
1.7	Review outcome goals	-	-	-	-	-	-	-	-	-	✓	-	*	-	*	*	-	-	-	-	-	-	4	1
Cluster Two: Feedback and monitoring																								
2.2	Feedback on behaviour	-	✓	✓	*	✓	-	✓	*	*	*	-	*	✓	✓	✓	-	✓	-	✓	*	-	15	9
2.3	Self-monitoring of behaviour	✓	✓	✓	✓	✓	-	✓	✓	✓	✓	-	-	✓	✓	✓	-	✓	✓	✓	✓	-	16	16
2.4	Self-monitoring of outcome(s) of behaviour	-	✓	✓	✓	-	-	✓	✓	✓	✓	-	✓	✓	✓	✓	-	✓	✓	✓	✓	-	15	15
2.7	Feedback on outcome(s) of behaviour	-	✓	-	-	-	-	-	-	-	-	-	-	-	-	-	-	-	-	-	-	-	1	1
Cluster Three: Social support																								
3.1	Social support (unspecified)	✓	-	✓	✓	-	-	-	*	*	✓	✓	✓	*	*	*	-	✓	-	✓	✓	-	14	9
3.2	Social support (practical)	-	-	-	-	-	-	-	-	-	-	✓	-	-	-	-	-	-	-	-	-	-	1	1
3.3	Social support (emotional)	-	-	-	*	-	-	-	✓	✓	-	-	-	*	-	-	-	*	-	-	*	-	6	2

No.	Behaviour Change Technique	Aguiar 2016	Arens 2018	Block 2015	Castro Sweet 2017	Cha 2014	Estabrooks 2008	Everett 2018	Fischer 2016	Fukuoka 2015	Kramer 2010	Limaye 2017	Ma 2013	Michaelides 2016	Piatt 2013 *DVD*	Piatt 2013 *Internet*	Ramachandran 2013	Sepah 2014	Tate 2003 *BI*	Tate 2003 *BeC*	Wilson 2017	Wong 2013	TOTAL (Inc. Imp.)	TOTAL (Exc. Imp.)
Cluster Four: Shaping knowledge																								
4.1	Instruction on how to perform the behaviour	✓	✓	-	-	-	-	-	-	-	-	✓	-	-	-	-	✓	-	-	-	-	-	4	4
4.2	Information about antecedents	-	-	-	*	-	-	-	-	*	-	✓	-	✓	-	-	-	*	-	-	*	-	6	2
Cluster Five: Natural consequences																								
5.1	Information about health consequences	✓	-	✓	-	-	-	-	-	-	-	✓	-	-	-	-	✓	-	-	-	-	✓	5	5
Cluster Six: Comparison of behaviour																								
6.1	Demonstration of the behaviour	✓	-	-	-	✓	-	-	-	-	-	-	-	-	-	-	-	-	-	-	-	-	2	2
6.2	Social comparison	-	-	✓	✓	-	-	-	*	*	-	-	-	*	-	-	-	✓	-	-	*	-	7	3
Cluster Seven: Associations																								
7.1	Prompts/cues	-	-	-	-	-	-	✓	-	-	*	-	*	-	*	✓	-	-	-	-	-	-	5	2
Cluster Eight: Repetition and substitution																								
8.2	Behaviour substitution	✓	-	-	-	-	-	-	-	-	-	✓	-	-	-	-	✓	-	-	-	-	-	3	3
8.3	Habit formation	✓	-	✓	-	-	-	-	-	-	-	-	-	-	-	-	-	-	-	-	-	-	2	2
8.4	Habit reversal	✓	-	-	-	-	-	-	-	-	-	-	-	-	-	-	-	-	-	-	-	-	1	1
8.7	Graded tasks	✓	-	-	-	-	-	-	-	-	-	-	-	-	-	-	-	-	-	-	-	-	1	1
Cluster Nine: Comparison of outcomes																								
9.1	Credible source	-	✓	✓	-	-	-	-	✓	-	✓	-	✓	-	✓	✓	-	-	-	-	-	-	7	7
Cluster Ten: Reward and threat																								
10.1	Material incentive (behaviour)	-	-	✓	-	-	-	-	-	-	-	-	-	-	-	-	-	-	-	-	-	-	1	1
10.2	Material reward (behaviour)	-	-	✓	-	-	-	-	-	-	-	-	-	-	-	-	-	-	-	-	-	-	1	1

No.	Behaviour Change Technique	Aguiar 2016	Arens 2018	Block 2015	Castro Sweet 2017	Cha 2014	Estabrooks 2008	Everett 2018	Fischer 2016	Fukuoka 2015	Kramer 2010	Limaye 2017	Ma 2013	Michaelides 2016	Piatt 2013 *DVD*	Piatt 2013 *Internet*	Ramachandran 2013	Sepah 2014	Tate 2003 *BI*	Tate 2003 *BeC*	Wilson 2017	Wong 2013	TOTAL (Inc. Imp.)	TOTAL (Exc. Imp.)
Cluster Eleven: Regulation																								
11.2	Reduce negative emotions	-	-	✓	-	-	-	-	-	-	-	✓	-	-	-	-	✓	-	-	-	-	-	3	3
Cluster Twelve Antecedents																								
12.3	Avoidance/reducing exposure to cues for the behaviour	-	-	-	-	-	-	-	-	-	-	✓	-	-	-	-	-	-	-	-	-	-	1	1
12.5	Adding objects to the environment	✓	✓	-	✓	-	-	-	-	-	✓	-	✓	-	✓	✓	-	✓	-	-	*	-	9	8
Cluster Fourteen: Scheduled consequences																								
14.4	Reward approximation	-	-	-	-	-	-	✓	-	-	-	-	-	-	-	-	-	-	-	-	-	-	1	1
TOTAL BCTs (including imputed BCTs)		13	3	10	11	10	11	6	2	10	13	10	10	10	13	13	5	5	2	2	11	1		
TOTAL BCTs (excluding imputed BCTs)		13	3	10	5	4	5	6	2	5	7	10	10	4	7	7	5	5	2	2	3	1		

Note: ✓BCT explicitly present; *BCT identified via imputation.

Supplementary Table 5.

Digital Features Identified in All Interventions

Digital feature	Aguiar (2016)	Arens (2018)	Block (2015)	Castro Sweet (2017)	Cha (2014)	Estabrooks (2008)	Everett (2018)	Fischer (2016)	Fukuoka (2015)	Kramer (2010)	Limaye (2017)	Ma (2013)	Michaelides (2016)	Piatt (2013) *DVD*	Piatt (2013) *Internet*	Ramachandran (2013)	Sepah (2014)	Tate (2003) *BI*	Tate (2003) *BeC*	Wilson (2017)	Wong (2013)	TOTAL
Passive features																						
Health/Lifestyle information and advice	✓	-	✓	-	-	✓	✓	✓	✓	✓	✓	*	✓	*	*	✓	-	✓	✓	-	✓	16
Activity tracking	✓	✓	✓	✓	✓	-	✓	-	✓	✓	-	✓	✓	✓	✓	-	✓	-	✓	*	-	15
Reminders and prompts	-	-	✓	✓	-	-	✓	✓	✓	-	-	✓	*	-	✓	-	✓	✓	-	*	-	11
Diet tracking	✓	-	✓	✓	✓	-	-	-	✓	-	-	✓	✓	-	-	-	✓	-	✓	✓	-	10
Weight and biomeasure tracking	-	✓	✓	✓	-	-	✓	-	✓	-	-	✓	✓	-	-	-	✓	-	-	*	-	9
Total passive features	3	2	5	4	2	1	4	2	5	2	1	5	5	2	3	1	4	2	3	4	1	
Interactive Features																						
Interactive health and lifestyle lessons	-	✓	✓	*	✓	-	-	-	✓	-	-	-	-	-	-	-	*	✓	✓	*	-	9
Online health coaching	-	✓	-	✓	-	-	-	-	-	-	-	✓	✓	-	✓	-	✓	-	✓	✓	-	8
Social media and support	-	-	✓	✓	-	-	-	-	-	-	✓	-	✓	-	-	-	✓	✓	✓	✓	-	8
Automated feedback	-	-	✓	-	-	✓	✓	-	✓	-	-	-	-	-	-	-	-	-	-	-	-	4
Gamification	-	-	✓	-	-	-	-	-	-	-	-	-	-	-	-	-	-	-	-	-	-	1
Total interactive features	0	2	4	3	1	1	1	0	2	0	1	1	2	0	1	0	3	2	3	3	0	
Total digital features	3	4	9	7	3	2	5	2	7	2	2	6	7	2	4	1	7	4	6	7	1	

Note: ✓Feature explicitly present; *Feature identified via imputation.

Supplementary Table 6.

Summary of Behaviour Change Technique Use in Effective and Non-Effective Interventions (Excludes Imputed BCTs)

No.	Behaviour Change Technique	All interventions (*N* = 21)		Effective ST (*N* = 12)		Not Effective ST (*N* = 7)		Effective LT (*N* = 4)		Not-Effective LT (*N* = 8)	
		n	%	*n*	%	*n*	%	*n*	%	*n*	%
Cluster One: Goals and planning											
1.1	Goal setting (behaviour)	8	38.1	4	33.3	3	42.9	1	25	2	25
1.2	Problem solving	9	42.9	4	33.3	3	42.9	2	50	2	25
1.3	Goal setting (outcome)	9	42.9	6	50	2	28.6	2	50	3	37.5
1.4	Action planning	4	19	3	25	1	14.3	1	25	0	0
1.5	Review behaviour goals	3	14.3	2	16.7	1	14.3	1	25	0	0
1.7	Review outcome goals	1	4.8	1	8.3	0	0	0	0	0	0
Cluster Two: Feedback and monitoring											
2.2	Feedback on behaviour	9	42.9	6	50	2	28.6	2	50	3	37.5
2.3	Self-monitoring of behaviour	16	76.2	11	91.7	4	57.1	3	75	6	75
2.4	Self-monitoring of outcome(s) of behaviour	15	71.4	11	91.7	3	42.9	4	100	6	75
2.7	Feedback on outcome(s) of behaviour	1	4.8	0	0	0	0	0	0	0	0
Cluster Three: Social support											
3.1	Social support (unspecified)	9	42.9	8	66.7	1	14.3	2	50	4	50
3.2	Social support (practical)	1	4.8	0	0	1	14.3	0	0	1	12.5
3.3	Social support (emotional)	2	9.5	1	8.3	1	14.3	0	0	1	12.5
Cluster Four: Shaping knowledge											
4.1	Instruction on how to perform the behaviour	4	19	1	8.3	1	14.3	0	0	1	12.5
4.2	Information about antecedents	2	9.5	1	8.3	1	14.3	1	25	1	12.5
Cluster Five: Natural consequences											
5.1	Information about health consequences	5	23.8	2	16.7	2	28.6	0	0	2	25

No.	Behaviour Change Technique	All interventions (*N* = 21)		Effective ST (*N* = 12)		Not Effective ST (*N* = 7)		Effective LT (*N* = 4)		Not-Effective LT (*N* = 8)	
		n	%	*n*	%	*n*	%	*n*	%	*n*	%
Cluster Six: Comparison of behaviour											
6.1	Demonstration of the behaviour	2	9.5	1	8.3	1	14.3	0	0	0	0
6.2	Social comparison	3	14.3	3	25	0	0	1	25	1	12.5
Cluster Seven: Associations											
7.1	Prompts/cues	2	9.5	1	8.3	1	14.3	1	25	0	0
Cluster Eight: Repetition and substitution											
8.2	Behaviour substitution	3	14.3	1	8.3	1	14.3	0	0	1	12.5
8.3	Habit formation	2	9.5	2	16.7	0	0	0	0	0	0
8.4	Habit reversal	1	4.8	1	8.3	0	0	0	0	0	0
8.7	Graded tasks	1	4.8	1		0	0	0	0	0	0
Cluster Nine: Comparison of outcomes											
9.1	Credible source	7	33.3	5	41.7	1	14.3	2	50	2	25
Cluster Ten: Reward and threat											
10.1	Material incentive (behaviour)	1	4.8	1	8.3	0	0	0	0	0	0
10.2	Material reward (behaviour)	1	4.8	1	8.3	0	0	0	0	0	0
Cluster Eleven: Regulation											
11.2	Reduce negative emotions	3	14.3	1	8.3	1	14.3	0	0	1	12.5
Cluster Twelve Antecedents											
12.3	Avoidance/reducing exposure to cues for the behaviour	1	4.8	0	0	1	14.3	0	0	1	12.5
12.5	Adding objects to the environment	8	38.1	7	58.3	0	0	3	75	2	25
Cluster Fourteen: Scheduled consequences											
14.4	Reward approximation	1	4.8	0	0	1	14.3	0	0	0	0
	Average number of BCTs per intervention	6.4		7.2		4.7		6.5		5	

Note: ST: short term (≤6 month) follow-up; LT: long term (≥12 month) follow-up. *N*: number of interventions; *n*: number of interventions in which the BCT was identified; %: proportion of interventions in each category that used the BCT.

Supplementary Table 7.

Summary of Digital Feature Use in Effective and Non-Effective Interventions (Excludes Imputed Features)

Digital features	All interventions (*N* = 21)		Effective ST (*N* = 12)		Not Effective ST (*N* = 7)		Effective LT (*N* = 4)		Not-Effective LT (*N* = 8)	
	n	%	*n*	%	*n*	%	*n*	%	*n*	%
Passive features										
Health and lifestyle information and advice	13	61.9	6	50	6	85.7	1	25	5	62.5
Activity tracking	14	66.7	11	91.7	2	28.6	4	100	3	37.5
Reminders and prompts	9	42.9	6	50	3	42.9	3	75	3	37.5
Diet tracking	10	47.6	9	75	1	14.3	3	75	3	37.5
Weight and biomeasure tracking	8	38.1	6	50	1	14.3	3	75	1	12.5
Average passive features per intervention	2.57 features		3.2 features		1.86 features		3.5 features		1.88 features	
Interactive features										
Interactive health and lifestyle lessons	6	28.6	3	25	2	28.6	0	0	2	25
Social media and support	8	38.1	6	50	2	28.6	2	50	5	62.5
Online health coaching	8	38.1	7	58.3	0	12.5	4	100	3	37.5
Automated feedback	4	19.0	2	16.7	2	28.6	0	0	0	0
Gamification	1	4.8	1	8.3	0	0	0	0	0	0
Average interactive features per intervention	1.29 features		1.58 features		0.86 features		1.5 features		1.25 feature	
Average total features per intervention	3.86		4.75		2.71		5		3.13	

Note: ST: short term (≤6 month) follow-up; LT: long term (≥12 month) follow-up. *N*: number of interventions; *n*: number of interventions in which the feature was identified; %: proportion of interventions in each category that used the digital feature.

Supplementary Table 8.

BCTs: Most Frequently Identified and Most Effective by Imputation vs No Imputation

BCTs most frequently identified in effective interventions (short term)	
With imputations included	With imputations excluded
Goal setting (behaviour)	Goal setting (behaviour)
Problem solving	Problem solving
Goal setting (outcome)	Goal setting (outcome)
Feedback on behavior	Feedback on behavior
Self-monitoring of behavior	Self-monitoring of behavior
Self-monitoring of outcome(s) of behavior	Self-monitoring of outcome(s) of behavior
Social support (unspecified)	Social support (unspecified)
Adding objects to the environment	Credible source
	Adding objects to the environment
**BCTs most frequently identified in effective interventions (long term)**	
With imputations included	With imputations excluded
Goal setting (behaviour)	Problem solving
Problem solving	Goal setting (outcome)
Goal setting (outcome)	Feedback on behavior
Feedback on behavior	Self-monitoring of behavior
Self-monitoring of behavior	Self-monitoring of outcome(s) of behavior
Self-monitoring of outcome(s) of behavior	Social support (unspecified)
Social support (unspecified)	Credible source
Adding objects to the environment	Adding objects to the environment
**Most effective BCTs (short term)**	
With imputations included	With imputations excluded
Social support (unspecified)	Social support (unspecified)
Adding objects to the environment	Adding objects to the environment
**Most effective BCTs (long term)**	
With imputations included	With imputations excluded
Problem solving	Adding objects to the environment

Note: The eight most frequently identified BCTs under each condition are listed. Nine BCTs are listed in the 'imputations excluded' column in the short term as two BCTs each registered the 8th highest frequency. The most effective BCTs were those identified at a considerably greater frequency in effective interventions versus non-effective interventions. All BCTs are listed in the order in which they appear in the BCT Taxonomy v1.

Supplementary Table 9.

Digital Features: Most Frequently Identified and Most Effective by Imputation vs No Imputation

Digital features most frequently identified in effective interventions (short term)	
With imputations included	With imputations excluded
Health and lifestyle info and advice (P)	Activity tracking (P)
Activity tracking (P)	Diet tracking (P)
Diet tracking (P)	Online health coaching (I)
**Digital features most frequently identified in effective interventions (long term)**	
With imputations included	With imputations excluded
Activity tracking (P)	Health and lifestyle info and advice (P)
Reminders and prompts (P)	Activity tracking (P)
Online health coaching (I)	Diet tracking (P)
	Online health coaching (I)
**Most effective digital features (short term)**	
With imputations included	With imputations excluded
Activity tracking (P)	Activity tracking (P)
Diet tracking (P)	Diet tracking (P)
Online health coaching (I)	
**Most effective digital features (long term)**	
With imputations included	With imputations excluded
Activity tracking (P)	Activity tracking (P)
Reminders and prompts (P)	Weight and biomeasure tracking (P)
Weight and biomeasure tracking (P)	Online health coaching (I)
Online health coaching (I)	

Note: P: passive feature; I: interactive feature
The three most frequently identified features under each condition are listed. Four digital features are listed in the 'imputations excluded' column in the long term as two features each registered the 3rd highest frequency. The most effective features were those identified at a considerably greater frequency in effective interventions versus non-effective interventions. All features are listed in the order in which they appear in the summary tables.

Supplementary Table 10.

Average Number of BCTs and Digital Features Used Per Intervention, both Including and Excluding Imputations

Average number of digital features used		
	Imputations included	Imputations excluded
All interventions (*N* = 21)		
Total features	4.3	3.86
Passive features	2.9	2.57
Interactive features	1.43	1.29
Effective short term (*N* = 12)		
Total features	5.58	4.75
Passive features	3.75	3.2
Interactive features	1.83	1.58
Not effective short term (*N* = 7)		
Total features	2.71	2.71
Passive features	1.86	1.86
Interactive features	0.86	0.86
Effective long term (*N* = 4)		
Total features	6	5
Passive features	4.25	3.5
Interactive features	1.75	1.5
Not effective long term (*N* = 8)		
Total features	3.88	3.13
Passive features	2.38	1.88
Interactive features	1.5	1.25

Average number of BCTs used		
	Imputations included	Imputations excluded
All interventions (*N* = 21)	9	6.4
Effective short term (*N* = 12)	11.3	7.2
Not effective short term (*N* = 7)	5.4	4.7
Effective long term (*N* = 4)	11.5	6.5
Not effective long term (*N* = 8)	7.8	3.7
