# Supplementary material for: A systematic review of the behaviour change techniques and digital features in technology-driven type 2 diabetes prevention interventions
Source: Digit Health. 2020 Mar 24;6:2055207620914427. doi: 10.1177/2055207620914427 (PMC7093696; doi:10.1177/2055207620914427)
Supplement: Supplementary material [file DHJ914427_Supplementary_Material5.pdf]

All Coded Digital and Non-digital Components

Key:

MoD = Mode of Delivery

SMS = Short Message Service (text message)

IVR = Interactive Voice Response

Web = Website

DVD = Digital Video Disc

SCU = Self-contained unit

App = Smartphone application

E/S = Email and SMS

W/E = Website and email

W/I = Website and IVR

A/S = Smartphone app and SMS

W/A = Website and smartphone app

S/E = SMS and email

Physical Per. = Physical Peripheral

*Denotes imputed components

Technological Components and Features				Study	Non-Technological Components	
Digital Component	MoD	Feature	Level		Format	Non-Digital Component
Food Information	Web	Health/Lifestyle Information	Passive	Aguiar (2014)	Hard Copy	Nutrition Education
Weight Loss Information	DVD	Health/Lifestyle Information	Passive		Hard Copy	Exercise Education
Pedometer	SCU	Activity Tracking	Passive		Hard Copy	Exercise Diary
Physical Activity Diary	Web	Activity Tracking	Passive		Hard Copy	Weight Log
Food Diary	Web	Diet Tracking	Passive		Hard Copy	Food Diary
					Hard Copy	Waist Circumferene Log
					Hard Copy	Resistance Training Guide
					Physical Per.	Tape Measure
					Physical Per.	Gymstick Resistance Band
Blood Glucose Log	App	Weight/Biomeasure Tracking	Passive	Arens (2018)	Face-to-Face	Nutrition Education
Blood Glucose Measuring Device	SCU	Weight/Biomeasure Tracking	Passive		Face-to-Face	Physical Activity Education
Weight Log	App	Weight/Biomeasure Tracking	Passive		Face-to-Face	Health Coaching
Waist Circumference Log	App	Weight/Biomeasure Tracking	Passive			
Blood Pressure Log	App	Weight/Biomeasure Tracking	Passive			
Pedometer	SCU	Activity tracking	Passive			
Online Health Coaching	App	Online Health Coaching	Interactive			
Attitudes Questionnaire	App	Health/Lifestyle lessons	Interactive			

Technological Components and Features				Study	Non-Technological Components	
Digital Component	MoD	Feature	Level		Format	Non-Digital Component
Push Notifications	App	Reminders/Prompts	Passive	Block (2015)		
Reminders	E/S	Reminders/Prompts	Passive			
Weight Log	Web	Weight/Biomeasure Tracking	Passive			
Activity Log	Web	Activity Tracking	Passive			
Diet Log	Web	Diet Tracking	Passive			
Links to External Resources	W/E	Health/Lifestyle Information	Passive			
Physical Activity Assessment	Web	Health/Lifestyle Lessons	Interactive			
Dietary Assessment	Web	Health/Lifestyle Lessons	Interactive			
Behavioural Strategy Lessons	Web	Health/Lifestyle Lessons	Interactive			
Diet Lessons	Web	Health/Lifestyle Lessons	Interactive			
Physical Activity Lessons	Web	Health/Lifestyle Lessons	Interactive			
Downloadable Worksheets	W/E	Health/Lifestyle Lessons	Interactive			
Online Points/Currency System	Web	Gamification	Interactive			
Team Challaneges	Web	Gamification	Interactive			
Social Message Board	Web	Social Media and Support	Interactive			
Family and Friend Referral System	Web	Social Media and Support	Interactive			
Automated Health Coaching	W/I	Automated Feedback	Interactive			
Weight Log	W/A	Weight/Biomeasure Tracking	Passive	Castro Sweet (2018)	Hard Copy	Resistance Exercise Guide*
Wireless Scale	SCU	Weight/Biomeasure Tracking	Passive		Hard Copy	Diet Log*
Pedometer	SCU	Activity Tracking	Passive		Physical Per.	Resistance Band*
Physical Activity Log	W/A	Activity Tracking	Passive		Physical Per.	Measuring Tape*
Diet Log	W/A	Diet Tracking	Passive			
Weight Notifications	W/A	Reminders/Prompts	Passive			
Health/Lifestyle Lessons*	W/A	Health/Lifestyle Lessons	Interactive			
Physical Activity Lessons*	W/A	Health/Lifestyle Lessons	Interactive			
Diet Lessons*	W/A	Health/Lifestyle Lessons	Interactive			
Online Social Forum	W/A	Social Media and Support	Interactive			
Online Health Coaching	W/A	Online Health Coaching	Interactive			

Technological Components and Features				Study	Non-Technological Components	
**Digital Component**	**MoD**	**Feature**	**Level**		**Format**	**Non-Digital Component**
Physical Activity Log	App	Activity Tracking	Passive	**Cha (2014)**	Face-to-Face	Nutrition Education
Food Log	App	Diet Tracking	Passive		Face-to-Face	Exercise Education
Homework Assignments	A/W	Health/lifestyle lessons	Interactive		Face-to-Face	Health Counseling
					Hard Copy	Health Summary
					Hard Copy	Educational Materials
Physical Activity Tips	IVR	Health/Lifestyle Information	Passive	**Estabrooks (2008)**	Face-to-Face	Healthy Lifestyle Education
Nutrition Tips	IVR	Health/Lifestyle Information	Passive			
Automated Diet Coaching	IVR	Automated Feedback	Interactive			
Automated PA Coaching	IVR	Automated Feedback	Interactive			
Weight Loss Advice	App	Health/Lifestyle Information	Passive	**Everett (2018)**	Face-to-Face	Lifestyle Counseling
Diet Advice	App	Health/Lifestyle Information	Passive		Hard Copy	T2D Prevention Information
Physical Activity Advice	App	Health/Lifestyle Information	Passive			
Digital Body Weight Scale	SCU	Weight/Biomeasure Tracking	Passive			
Weight Log	App	Weight/Biomeasure Tracking	Passive			
Weight Notifications	App	Reminders/Prompts	Passive			
Personalised Push Notifications	App	Reminders/Prompts	Passive			
Physical Activity Log	App	Activity Tracking	Passive			
Automated Physical Activity Coaching	App	Automated Feedback	Interactive			
Health/lifestyle Advice	SMS	Health/Lifestyle Information	Passive	**Fischer (2016)**	Face-to-Face	Diet Support
Physical Activity Advice	SMS	Health/Lifestyle Information	Passive		Face-to-Face*	Health/Lifestyle Education*
Nutrition Advice	SMS	Health/Lifestyle Information	Passive		Face-to-Face*	Physical Activity Education*
Links to Additional Resources	SMS	Health/Lifestyle Information	Passive		Face-to-Face*	Nutrition Education*
Weight Report Reminders	SMS	Reminders/Prompts	Passive		Phone	Motivational Interviewing

Technological Components and Features				Study	Non-Technological Components	
Digital Component	MoD	Feature	Level		Format	Non-Digital Component
Health/lifestyle Videos	App	Health/Lifestyle Information	Passive	Fukuoka (2015)	Face-to-Face	Health/Lifestyle Education
Physical Activity Videos	App	Health/Lifestyle Information	Passive		Face-to-Face	Physical Activity Education
Diet Videos	App	Health/Lifestyle Information	Passive		Face-to-Face	Diet Education
Pedometer	A/S	Activity Tracking	Passive			
Physical Activity Log	App	Activity Tracking	Passive			
Reminders	A/S	Reminders/Prompts	Passive			
Weight Log	App	Weight/Biomeasure Tracking	Passive			
Diet Log	App	Diet Tracking	Passive			
Auto Self-Monitoring Feedback	App	Automated Feedback	Interactive			
Health/Lifestyle Messages	App	Automated Feedback	Interactive			
Physical Activity Messages	App	Automated Feedback	Interactive			
Diet Messages	App	Automated Feedback	Interactive			
Health/Lifestyle Quizzes	App	Health/Lifestyle Lessons	Interactive			
Physical Activity Quizzes	App	Health/Lifestyle Lessons	Interactive			
Diet Education Quizzes	App	Health/Lifestyle Lessons	Interactive			
Health/Lifestyle Education Videos	DVD	Health/Lifestyle Information	Passive	Kramer (2010)	Hard Copy	Health/Lifestyle Worksheets
Physical Activity Education Videos	DVD	Health/Lifestyle Information	Passive		Hard Copy	Physical Activity Worksheets
Nutrition Education Videos	DVD	Health/Lifestyle Information	Passive		Hard Copy	Nutrition Worksheets
Pedometer	SCU	Activity Tracking	Passive		Hard Copy	Weight Log*
					Hard Copy	Physical Activity Log
					Hard Copy	Diet Log
					Hard Copy	Pedometer Guide*
					Hard Copy	Resistance Training Guide*
					Hard Copy	Stretching Guide*
					Physical Per.	Resistance Bands*
					Phone	Remote Health Coaching

Technological Components and Features				Study	Non-Technological Components	
Digital Component	MoD	Feature	Level		Format	Non-Digital Component
Lifestyle Advice	SM/E	Health/Lifestyle Information	Passive	**Limaye (2017)**	Hard Copy	Diet Information
Physical Activity Advice	SM/E	Health/Lifestyle Information	Passive		Hard Copy	Physical Activity Information
Diet Advice	SM/E	Health/Lifestyle Information	Passive		Face-to-Face	Lifestyle Modification Class
Stress Managament Advice	SM/E	Health/Lifestyle Information	Passive			
Facebook Group	Web	Social Media and Support	Interactive			
Health/Lifestyle Education Videos*	DVD	Health/Lifestyle Information	Passive	**Ma (2013)**	Hard Copy	Health/lifestyle worksheets*
Physical Activity Education Videos*	DVD	Health/Lifestyle Information	Passive		Hard Copy	Physical activity worksheets*
Nutrition Education Videos*	DVD	Health/Lifestyle Information	Passive		Hard Copy	Nutrition Worksheets*
Pedometer	SCU	Activity Tracking	Passive		Hard Copy	Pedometer Guide*
Physical Activity Log	Web	Activity Tracking	Passive		Hard Copy	Resistance Training Guide*
Reminders	Email	Reminders/Prompts	Passive		Hard Copy	Stretching Guide*
Weight Log	Web	Weight/Biomeasure Tracking	Passive		Physical Per.	Resistance Bands*
Diet Log	Web	Diet Tracking	Passive		Physical Per.	Weight Scale
Online Health Coaching	Web	Online Health Coaching	Interactive			
Health/Lifestyle Education	App	Health/Lifestyle Information	Passive	**Michaelides (2016)**	Phone	Remote Healh Counseling
Physical Activity Education	App	Health/Lifestyle Information	Passive			
Diet Education	App	Health/Lifestyle Information	Passive			
Health/Lifestyle Challenges*	App	Reminders/Prompts	Passive			
Physical Activity Challenges*	App	Reminders/Prompts	Passive			
Diet Challeneges*	App	Reminders/Prompts	Passive			
Weight Log	App	Weight/Biomeasure Tracking	Passive			
Physical Activity Log	App	Activity Tracking	Passive			
Diet Log	App	Diet Tracking	Passive			
Online Health Coaching	App	Online Health Coaching	Interactive			
Group Messaging	App	Social Media and Support	Interactive			

Technological Components and Features				Study	Non-Technological Components	
**Digital Component**	**MoD**	**Feature**	**Level**		**Format**	**Non-Digital Component**
Health/Lifestyle Education Videos*	DVD	Health/Lifestyle Information	Passive	**Piatt (2013)** *DVD*	Hard Copy	Health/Lifestyle Worksheets
Physical Activity Education Videos*	DVD	Health/Lifestyle Information	Passive		Hard Copy	Physical Activity Worksheets
Nutrition Education Videos*	DVD	Health/Lifestyle Information	Passive		Hard Copy	Nutrition Worksheets
Pedometer	SCU	Activity Tracking	Passive		Hard Copy	Weight Log
					Hard Copy	Physical Activity Log
					Hard Copy	Diet Log
					Hard Copy	Pedometer Guide*
					Hard Copy	Resistance Training Guide*
					Hard Copy	Stretching Guide*
					Physical Per.	Resistance Bands*
					Physical Per.	Measuring Cups and Spoons
					Phone	Remote Health Coaching
					Face-to-Face	Group Debriefing Sessions
Health/Lifestyle Education Videos*	Web	Health/Lifestyle Information	Passive	**Piatt (2013)** *Internet*	Hard Copy	Health/Lifestyle Worksheets
Physical Activity Education Videos*	Web	Health/Lifestyle Information	Passive		Hard Copy	Physical Activity Worksheets
Nutrition Education Videos*	Web	Health/Lifestyle Information	Passive		Hard Copy	Nutrition Worksheets
Pedometer	SCU	Activity Tracking	Passive		Hard Copy	Weight Log
Reminders/prompts	Email	Reminders/Prompts	Passive		Hard Copy	Physical Activity Log
Online Counseling	Web	Online Health Coaching	Interactive		Hard Copy	Diet Log
					Hard Copy	Pedometer Guide*
					Hard Copy	Resistance Training guide*
					Hard Copy	Stretching Guide*
					Physical Per.	Resistance Bands*
					Physical Per.	Measuring Cups and Spoons

Technological Components and Features				Study	Non-Technological Components	
Digital Component	MoD	Feature	Level		Format	Non-Digital Component
Healty Lifestyle Education	SMS	Health/Lifestyle Information	Passive	**Ramachandran (2013)**	Hard Copy	Diet Information
Diet Information	SMS	Health/Lifestyle Information	Passive		Hard Copy	Physical Activity Information
Physical Activity Information	SMS	Health/Lifestyle Information	Passive		Face-to-Face	Healty Lifestyle Education
Wireless Scale	SCU	Weight/Biomeasure Tracking	Passive	**Sepah (2014)**	Hard Copy	Resistance Exercise Guide*
Weight Log	W/A	Weight/Biomeasure Tracking	Passive		Hard Copy	Diet Log*
Pedometer	SCU	Activity Tracking	Passive		Physical Per.	Resistance Band*
Physical Activity Log	W/A	Activity Tracking	Passive		Physical Per.	Measuring Tape*
Diet Log	W/A	Diet Tracking	Passive		Physical Per.	Photo Frame
Weight Notifications	W/A	Reminders/Prompts	Passive			
Health/Lifestyle Lessons*	W/A	Health/Lifestyle Lessons	Interactive			
Physical Activity Lessons*	W/A	Health/Lifestyle Lessons	Interactive			
Diet Lessons*	W/A	Health/Lifestyle Lessons	Interactive			
Online Social Forum	W/A	Social Media and Support	Interactive			
Online Health Coaching	W/A	Online Health Coaching	Interactive			
Weight Loss Tips	Web	Health/Lifestyle Information	Passive	**Tate (2003)** *Basic Internet*	Face-to-Face	Diet Information
Weight Loss Resources	Web	Health/Lifestyle Information	Passive		Face-to-Face	Exercise Information
Weight Submission Reminders	Email	Reminders/Prompts	Passive		Face-to-Face	Behaviour Change Information
Message Board	Web	Social Media and Support	Interactive		Hard Copy	Diet Log
Weight Loss Tutorials	Web	Health/Lifestyle Lessons	Interactive		Hard Copy	Exercise Log
Weight Loss Tips	Web	Health/Lifestyle Information	Passive	**Tate (2003)** *Behavioural e-Counseling*	Face-to-Face	Diet Information
Weight Loss Resources	Web	Health/Lifestyle Information	Passive		Face-to-Face	Exercise Information
Food Diary	Web	Diet Tracking	Passive		Face-to-Face	Behaviour Change Information
Exercise Diary	Web	Activity Tracking	Passive		Hard Copy	Diet Log
Message Board	Web	Social Media and Support	Interactive		Hard Copy	Exercise Log
Weight Loss Tutorials	Web	Health/Lifestyle Lessons	Interactive			
Remote Health Coaching	Email	Online Health Coaching	Interactive			

Technological Components and Features				Study	Non-Technological Components	
Digital Component	MoD	Feature	Level		Format	Non-Digital Component
Wireless Scale	SCU	Weight/Biomeasure Tracking	Passive	**Wilson (2017)**	Hard Copy	Resistance Exercise Guide*
Weight Log	W/A	Weight/Biomeasure Tracking	Passive		Hard Copy	Diet Log*
Pedometer*	SCU	Activity Tracking	Passive		Physical Per.	Resistance Band*
Physical Activity Log	W/A	Activity Tracking	Passive		Physical Per.	Measuring Tape*
Diet Log	W/A	Diet Tracking	Passive			
Weight Notifications*	W/A	Reminders/Prompts	Passive			
Health/Lifestyle Lessons*	W/A	Health/Lifestyle Lessons	Interactive			
Physical Activity Lessons*	W/A	Health/Lifestyle Lessons	Interactive			
Diet Lessons*	W/A	Health/Lifestyle Lessons	Interactive			
Online Social Forum	W/A	Social Media and Support	Interactive			
Online Health Coaching	W/A	Online Health Coaching	Interactive			
T2D/Prediabetes Information	SMS	Health/Lifestyle Information	Passive	**Wong (2013)**	Hard Copy	T2D/Prediabetes Information
Lifestyle Modification Information	SMS	Health/Lifestyle Information	Passive		Hard Copy	Health Behaviour Information
