# Supplementary material for: A systematic review of the behaviour change techniques and digital features in technology-driven type 2 diabetes prevention interventions
Source: Digit Health. 2020 Mar 24;6:2055207620914427. doi: 10.1177/2055207620914427 (PMC7093696; doi:10.1177/2055207620914427)
Supplement: Supplementary material [file DHJ914427_Supplementary_Material6.pdf]

Digital Feature Descriptions

Passive Features

Health and Lifestyle Information and Advice. This includes health and lifestyle-related information, advice, and educational materials. These may come in the form of information on how to eat healthily and increase physical activity; how to reduce stress; and how to make lifestyle changes to improve overall health, lose weight, or reduce the risk of developing type 2 Diabetes. This feature only includes information and advice that can be read (e.g., via website) or viewed (e.g., via online video or DVD) and does not include structured, interactive lessons or quizzes.

Activity Tracking. This includes any digital tool used to count, track, and/or record physical activity behaviours. Such tools include: pedometers, accelerometers, and digital physical activity logs. These tools may require participants to enter their own data (e.g., steps per day, exercise session duration), or they may record the data automatically. Activity tracking is a passive feature, as although participants use these tools to record data, they offer one-way interaction with no return feedback. However, if for example, a smartphone application automatically tracked a participant's daily steps and the participant automatically received feedback on their performance (e.g., messages of praise and encouragement and/or adjustment of the daily step goal), the application would contain two features: activity tracking, and automated feedback. This two-feature scenario also applies to diet tracking, and weight and biomeasure tracking.

Reminders and Prompts. This describes any one-way message or notification sent to participants with the purpose of reminding them to complete a specific action or task. Forms can include text messages, alarms, push notifications, and calendar reminders either sent by a health coach or delivered automatically. The types of notifications may include: reminding a participant to weigh themselves, eat five servings of fruit and vegetables, drink water, or submit their tracking logs. These reminders and prompts are part of the intervention protocol and do not include cases where participants set their own reminders.

Diet Tracking. This includes any digital tool used to count, track, and/or record dietary behaviours. Diet tracking tools can include: calorie counters, food diaries, and digital food scales. These tools mostly require participants to enter their own data such as the number of servings of foods and beverages consumed each day, and/or daily caloric intake.

Weight and Biomeasure Tracking. This includes any digital tool used to count, track, and/or record body weight or other biological outcome measures such as blood glucose. These tools include wireless digital body weight scales, digital blood glucose monitors, and digital diaries that are used to track outcome data.

Interactive Features

Interactive Health and Lifestyle Lessons. This feature includes interactive educational sessions such as lessons, tutorials, or quizzes in which participants read or view health and lifestyle information and advice as described above, and then respond in the form of assignments, case studies, or quiz responses. Feedback on these responses may or may not be given.

Social Media and Support. This includes any digital tool that either enables participants to interact with others (e.g., other participants, friends, family members) socially, or that is used by participants to seek social support. This includes Facebook groups, online message boards or chat rooms, peer-to-peer instant messaging, and online referral tools to share the intervention content with others. This does not include digitally-facilitated interactions with a health coach as this would fall under the online health coaching feature.

Automated Feedback. This feature describes automated two-way behavioural and lifestyle support. Feedback is automatically generated, based on participants' action(s) or reported data. This can be facilitated by Interactive Voice Response (IVR), automated text message, and smartphone application. For example, in IVR, a participant receives an automated telephone call that provides a range of health messages or lifestyle tips. Participants then have the option to select a specific message or provide an alternative response to the message via the phone's keypad. The IVR system then tailors the next call or tip based on the participant's previous response.

Gamification. For this review, gamification refers to any digital component that was used as a game or part of a game to add fun or challenge to the intervention whilst providing explicit incentive or reward. An example would be the use of an online points system. Here, participants may earn digital points each time they complete a specific action or series of tasks. These points may be used as friendly competition among participants, or they may be redeemed for actual prizes and rewards.
