# Supplementary material for: A systematic review of the behaviour change techniques and digital features in technology-driven type 2 diabetes prevention interventions
Source: Digit Health. 2020 Mar 24;6:2055207620914427. doi: 10.1177/2055207620914427 (PMC7093696; doi:10.1177/2055207620914427)
Supplement: Supplementary material [file DHJ914427_Supplementary_Material7.pdf]

Supplementary material for:

A Systematic Review of the Behaviour Change Techniques and Digital Features in Technology-Driven Type 2 Diabetes Prevention Interventions.

Luke Van Rhoon[a*], Molly Byrne[a], Eimear Morrissey[a], Jane Murphy[b], and Jenny McSharry[a]

Supplementary File 1: PRISMA checklist

Supplementary File 2: Search strategy

Supplementary File 3: References for all included articles

Supplementary File 4: Supplementary tables 1-10

Table 1. Quality Assessment Summaries for All Studies
Table 2. The Proportion of Baseline Weight Lost at Each Follow-Up for Interventions Included in the Primary Effectiveness Analysis
Table 3. Changes in Body Weight and Glycaemia from Baseline to Most Recent Follow-up (Expressed in Mean Values Unless Otherwise Indicated)
Table 4. Behaviour Change Techniques Identified in All Interventions
Table 5. Digital Features Identified in All Interventions
Table 6. Summary of Behaviour Change Technique Use in Effective and Non-Effective Interventions (Excludes Imputed BCTs)
Table 7. Summary of Digital Feature Use in Effective and Non-Effective Interventions (Excludes Imputed Features)
Table 8. BCTs: Most Frequently Identified and Most Effective by Imputation vs No Imputation
Table 9. Digital Features: Most Frequently Identified and Most Effective by Imputation vs No Imputation
Table 10. Average Number of BCTs and Digital Features Used Per Intervention, both Including and Excluding Imputations

Supplementary File 5

All coded digital and non-digital components

Supplementary File 6

Digital feature descriptions
